# Cancer cell plasticity: from cellular, molecular, and genetic mechanisms to tumor heterogeneity and drug resistance

**DOI:** 10.1007/s10555-024-10172-z

**Published:** 2024-02-08

**Authors:** Gh Rasool Bhat, Itty Sethi, Hana Q. Sadida, Bilal Rah, Rashid Mir, Naseh Algehainy, Ibrahim Altedlawi Albalawi, Tariq Masoodi, Gowtham Kumar Subbaraj, Farrukh Jamal, Mayank Singh, Rakesh Kumar, Muzafar A. Macha, Shahab Uddin, Ammira S. Al-Shabeeb Akil, Mohammad Haris, Ajaz A. Bhat

**Affiliations:** 1 Advanced Centre for Human Genetics, Sher-I-Kashmir Institute of Medical Sciences, Soura, Srinagar, Jammu and Kashmir India; 2 Institute of Human Genetics, University of Jammu, Jammu, Jammu and Kashmir India; 3 Department of Human Genetics-Precision Medicine in Diabetes, Obesity and Cancer Program, Sidra Medicine, Doha, Qatar; 4 Iron Biology Group, Research Institute of Medical and Health Science, University of Sharjah, Sharjah, UAE; 5 Department of Medical Laboratory Technology, Faculty of Applied Medical Sciences, Prince Fahad Bin Sultan Chair for Biomedical Research, University of Tabuk, Tabuk, Saudi Arabia; 6 Department of Surgical Oncology, Faculty of Medicine, University of Tabuk, Tabuk, Saudi Arabia; 7 Laboratory of Cancer Immunology and Genetics, Sidra Medicine, Doha, Qatar; 8 Chettinad Hospital and Research Institute, Kelambakkam, Tamil Nadu India; 9 Dr. Rammanohar, Lohia Avadh University, Ayodhya, India; 10 Department of Medical Oncology (Lab.), Institute of Medical Sciences (AIIMS), Dr. BRAIRCH, All India New Delhi, India; 11 School of Biotechnology, Shri Mata Vaishno Devi University, Katra, Jammu and Kashmir India; 12 Watson-Crick Centre for Molecular Medicine, Islamic University of Science and Technology, Awantipora, Jammu and Kashmir India; 13 Translational Research Institute, Academic Health System, Hamad Medical Corporation, Doha, Qatar; 14 Laboratory Animal Research Centre, Qatar University, Doha, Qatar; 15 Center for Advanced Metabolic Imaging in Precision Medicine, Department of Radiology, Perelman School of Medicine, University of Pennsylvania, Philadelphia, USA

**Keywords:** Cancer cell plasticity, Cancer stem cells, Epithelial-mesenchymal transition, Tumor heterogeneity, Drug resistance, Targeted therapies

## Abstract

Cancer is a complex disease displaying a variety of cell states and phenotypes. This diversity, known as cancer cell plasticity, confers cancer cells the ability to change in response to their environment, leading to increased tumor diversity and drug resistance. This review explores the intricate landscape of cancer cell plasticity, offering a deep dive into the cellular, molecular, and genetic mechanisms that underlie this phenomenon. Cancer cell plasticity is intertwined with processes such as epithelial-mesenchymal transition and the acquisition of stem cell–like features. These processes are pivotal in the development and progression of tumors, contributing to the multifaceted nature of cancer and the challenges associated with its treatment. Despite significant advancements in targeted therapies, cancer cell adaptability and subsequent therapy-induced resistance remain persistent obstacles in achieving consistent, successful cancer treatment outcomes. Our review delves into the array of mechanisms cancer cells exploit to maintain plasticity, including epigenetic modifications, alterations in signaling pathways, and environmental interactions. We discuss strategies to counteract cancer cell plasticity, such as targeting specific cellular pathways and employing combination therapies. These strategies promise to enhance the efficacy of cancer treatments and mitigate therapy resistance. In conclusion, this review offers a holistic, detailed exploration of cancer cell plasticity, aiming to bolster the understanding and approach toward tackling the challenges posed by tumor heterogeneity and drug resistance. As articulated in this review, the delineation of cellular, molecular, and genetic mechanisms underlying tumor heterogeneity and drug resistance seeks to contribute substantially to the progress in cancer therapeutics and the advancement of precision medicine, ultimately enhancing the prospects for effective cancer treatment and patient outcomes.

## Introduction

Cancer stands as one of the most formidable health challenges of our time, driven by the unrestrained growth and division of abnormal cells within the body. This unwarranted proliferation infiltrates and damages healthy tissues, culminating in a diverse spectrum of disorders, each identified by the cell or tissue type of origin. With a projected statistic that one in every four individuals over the age of 65 will have battled cancer by 2040, it is underscored as a leading global cause of death [1].

This multifaceted disease evolves through numerous steps, with many malignant pathways leading to diverse tumor types and subtypes. Each pathway includes unique aberrations and consequentially acquired traits necessary for overcoming tissue-specific barriers in specific tumorigenesis pathways [2, 3].

However, one critical aspect threading through this complexity is cancer cell plasticity. This phenomenon allows cancer cells to adapt and change, making them even more resilient and difficult to treat. Cancer cell plasticity is closely related to the epithelial-mesenchymal transition and the acquisition of stem cell features, both of which play significant roles in tumor development, diversity, and treatment resistance. Understanding this plasticity is crucial for developing more effective therapeutic strategies, as it lies at the heart of cancer’s adaptability and consequent resistance to treatment. Despite the extensive range, the prevalence of cancer cell plasticity remains a consistent, underlining theme, integral for metastasis and contributing to intratumoral heterogeneity [4].

The cellular plasticity empowers tumor cells to modify their phenotypes, facilitating their evasion from terminal differentiation. This characteristic significantly hampers effective cancer management by bolstering tumor response modification and inducing therapy resistance. It engages cellular programs like epithelial-to-mesenchymal transition (EMT) and influences significant cell signaling pathways, including Wnt and Notch, underscoring the role of phenotypic plasticity in tumor development and diversity [5].

The deduction of mechanisms underlying dedifferentiation and transdifferentiation, prompted by the loss of specific developmental transcription factors and the emergence of stem and progenitor characteristics, underscores the significance of understanding this plasticity for enhanced therapeutic strategies [6–8].

Beyond phenotypic plasticity, the epigenetic landscape also plays a pivotal role in cancer’s menacing dance. The emergence of non-mutational epigenetic reprogramming presents a parallel path to cancer development, aside from genetic mutations [9–11]. This mechanism underscores the profound impact of the tumor microenvironment, such as hypoxia-induced methylation changes, reflecting the intricate interplay between various factors influencing cancer progression [12]. Navigating the cancer labyrinth demands a profound understanding of its multiple facets, from the genetic and cellular levels to the interaction with the broader microenvironment. The embrace of the hallmarks of cancer, which epitomizes the acquired capabilities of cancer cells, serves as a beacon in this endeavor. Recent advancements have further expanded these hallmarks to include the deregulation of cellular metabolism and evasion from immune destruction, underscoring the continuous evolution of our understanding of this complex disease.

In conclusion, this comprehensive perspective on cancer, from cellular plasticity to epigenetic reprogramming, emphasizes the necessity for innovative and effective therapeutic approaches. A comprehensive understanding of cancer mechanisms and hallmarks can lead to more effective therapeutic strategies and bring us closer to conquering this global malady. This review thoroughly explores the complex realms of cellular plasticity, delving into the underlying cellular, molecular, and epigenetic mechanisms. This review sheds light on the complex interaction of mechanisms that collectively contribute to the dynamic phenomenon of cellular plasticity. By elucidating the role of tumor heterogeneity in inducing plasticity, this examination highlights the diverse pathways through which cellular alterations transpire, leading to varying and often unpredictable cellular responses.

Particular emphasis is placed on the critical significance of cellular plasticity in the emergence of drug resistance. The review dissects the intricate roles of different forms of cell plasticity, including transitions from temporary drug-tolerant states to irreversible drug resistance, providing valuable insights into the challenges and opportunities in targeting plasticity for enhanced therapeutic outcomes. The detailed analysis presented in this review paves the way for identifying potential biomarkers and developing innovative strategies to counteract the debilitating impact of cellular plasticity on treatment efficacy.

Beyond merely presenting existing knowledge, the review propels the conversation forward by considering the unresolved questions and potential directions for future research. It advocates for a robust, multifaceted approach to studying and targeting cellular plasticity, underscoring the necessity for continuous innovation and collaboration in the relentless pursuit of more effective and sustainable cancer therapies.

By delving into the cellular, molecular, and epigenetic underpinnings of cellular plasticity and highlighting the compelling link between tumor heterogeneity, cellular plasticity, and drug resistance, this review stands as a pivotal resource for researchers and clinicians alike, fostering a deeper understanding and offering a foundation for future advancements in cancer therapeutics.

## Cellular mechanisms of cancer cell plasticity

Phenotypic plasticity is critical to the genesis, development, and therapeutic outcomes of cancer at the cellular level. While strides are being made in pinpointing the key factors that govern the shift from hierarchical organization to phenotypic plasticity within cellular structures, there remains an urgent need for more profound insights into the specific signatures and underlying mechanisms that orchestrate either transdifferentiation or dedifferentiation phases. Moreover, the role of phenotypic plasticity in spawning diversity both within and between tumors is an area that continues to elude our comprehensive understanding. Intriguingly, the very nature of phenotypic plasticity could present unforeseen vulnerabilities within cancer’s complex biological landscape. Recognizing this, it becomes crucial to explore the potential of leveraging these aspects of plasticity for the development of innovative anticancer therapeutics [13].

Cellular differentiation stages in healthy cells and tissues are dynamically controlled by activating or inactivating certain transcriptional factors. Because both variables contribute to the abnormal activation of developmental programs, the factors that encourage cellular plasticity during development and wound healing also produce phenotypic plasticity in cancer [14].

### Epithelial-mesenchymal transition

EMT represents a specific form of transdifferentiation, where cancer cells undergo a reversible shift from an epithelial to a mesenchymal phenotype, a process driven not by genetic alterations but by epigenetic modifications [15]. This complex transformation is marked by the loss of apical-basal polarity, a breakdown of intracellular junctions, the emergence of front-rear polarity, and extensive remodeling of the cytoskeleton. Conversely, the mesenchymal-epithelial transition (MET) serves as the reverse process of EMT [16–18]. Central to the regulatory networks of both EMT and MET are EMT-inducing transcription factors (EMT-TFs) such as Snail, Slug, Zeb1/Zeb2, and Twist, as well as specific microRNAs (miRs). These elements are believed to hold considerable sway over the transcriptional shifts characterizing these transitions. Moreover, key signaling pathways, including transforming growth factor (TGF)-β, Wingless/Integrated (WNT), Notch, and Hippo, have been identified as significant contributors to the processes [19–21]. Their involvement underscores the intricate molecular interplay at work during these cellular metamorphoses.

The Snail, zinc finger E-box-binding homeobox (ZEB), and Twist families embody master transcription factors (TFs) known for orchestrating transcriptional networks that induce dedifferentiation, standing out among the extensively researched mechanisms of cellular plasticity. These TFs are pivotal in recognizing and binding to specific DNA sequences, thereby initiating precise genetic programs. Snail family proteins, including Snail/SNAI1, Slug/SNAI2, and Smuc/SNAI3, are characterized by their zinc finger domains and function primarily as transcriptional repressors. These elements, conserved across vertebrates, are integral to a plethora of cellular functions and developmental milestones. They play influential roles in processes as diverse as mesoderm formation, neural crest migration, establishment of left–right body asymmetry, regulation of cell motility, and apoptosis. Furthermore, their implication extends to critical stages in the genesis and advancement of cancer [22–24]. In the same spectrum, zinc finger E-box-binding homeobox 1 (ZEB1) and zinc finger E-box-binding homeobox 2 (ZEB2) represent key components of the ZEB family, distinguished by their unique structural configuration of dual zinc finger clusters that are notably separated, with a conserved homeodomain nestled between them. These zinc finger proteins are not just architectural curiosities; they have profound implications for the transcriptional regulation integral to cellular processes and phenotypic plasticity. Their role, although subtly refined, is fundamental in the grand schema of developmental biology and oncogenesis.

Despite its resemblance to the POU homeodomain, the homeodomain within the ZEB family of TFs does not directly engage with DNA. Instead, it is speculated to contribute to protein–protein interactions, indicating a more nuanced role in cellular regulatory mechanisms. Much like their counterparts in the Snail family, ZEB-TFs execute transcriptional repression, but they do so through an epigenetic approach that selectively targets specific DNA regions. ZEB1 and ZEB2 are particularly noteworthy for their PXDLS motifs, which serve as docking sites for epigenetic silencing assemblies such as the CtBP core complex 2 and co-RE1 silencing transcription factor (coREST). This strategic interaction facilitates the imposition of repressive histone marks, effectively muting the expression of ZEB target genes [25]. The process underscores a sophisticated layer of genetic regulation, where transcription factors enact their roles not just through direct DNA binding but through the intricate ballet of protein interactions and epigenetic modifications.

Beyond the core EMT-TFs, a diverse array of additional TFs, including TBXT, E47, KLF4, PPRX1, GSC, RUNX1, TCF4, SIX1, FOXC2, and SOX4, can also initiate EMT. The expression profiles of these factors vary significantly based on the tissue type or nature of the malignancy involved [26]. At this juncture, miRs play a crucial regulatory role. For instance, miR-200 directly represses ZEB-TFs, while miR-34 and miR-200 curtail SNAI2/SLUG, and miR-203 targets SNAI1 [27]. These transcription factors have nuanced roles: they can inhibit the expression of epithelial markers, particularly CDH1 and CRB3, while simultaneously promoting components of epithelial junctions. Conversely, they suppress mesenchymal gene expression, including genes like VIM, FN1, and CDH2, and elevate the levels of proteolytic enzymes, such as metalloproteinases, alongside various cytoskeletal proteins [27]. Malignant tissues induce EMT programs through autocrine or paracrine pathways, involving a diverse range of growth factors beyond the well-documented TGF, such as EGF, HGF, FGF, VEGF, and IGF [28], as well as cytokines like IL-8. Furthermore, environmental cues like hypoxia, mechanical stress from the extracellular matrix (ECM), or the presence of specific oncometabolites can powerfully trigger EMT.

Interestingly, the tumor immune microenvironment, marked by IFN-regulated genes such as FN1 and CRB3, might also influence EMT programs [26, 28]. This complex interplay highlights the multifaceted nature of EMT regulation, where cellular, molecular, and environmental factors converge to dictate cellular identity and behavior.

### Mesenchymal-epithelial transition

Cancer cells have a remarkable capacity for phenotypic plasticity, which enables them to acquire many biological states as a tumor develops. Cancer cell plasticity is one such process that leads to a variety of tumors and treatment resistance. MET is a critical biological phenomenon that reverses EMT. Dynamic cellular transitions, particularly the change from sessile epithelial to motile mesenchymal states, are crucial for embryogenesis and organogenesis because they enable cells to change their morphology and how they interact with the extracellular environment and other cells. EMT plays a crucial role in gastrulation during development, which controls the establishment of the primitive streak that later determines the body layout. Equally significant, MET happens several times throughout embryogenesis. As the trophectoderm develops after implantation, the first embryonic epithelium’s precursor cells are produced [29]. This is the earliest instance of MET. Kidney organogenesis and somitogenesis have been the most well researched [30, 31]. However, cellular transitions are not just present during embryogenesis, and there have been multiple examples of EMT/MET in adult tissues due to misusing these essential embryological processes. As an illustration, adult tissues frequently include mesenchymal stem cells, which provide a pool of cells that may differentiate into either mesenchymal or epithelial derivatives [32].

As carcinoma develops and progresses, mounting evidence shows that cells’ ability to undergo cellular transitions is highly advantageous. EMT, for example, explains the remarkable process that allows cancer cells derived from epithelial tissues to adopt a migratory and invasive phenotype, facilitating escape from the primary site and encouraging the development of metastases [33]. The preservation of the mesenchymal phenotype is not necessary and may even be restricted to the formation of occult metastases at the metastatic site. It may be preferable to return to the MET phenotype, which is an epithelial phenotype.

Although several signaling pathways and transcription factors orchestrate MET, their dysregulation can result in the development of a mesenchymal phenotype that is either reversible or not [34]. TGF-β, Wnt, Notch, and receptor tyrosine kinases (RTKs) are some of the molecular regulators of MET. For instance, TGF signaling, which is frequently activated in cancer, controls both EMT and MET. Additionally, transcription factors known for supporting EMT, such as Snail, ZEB, and Twist, can block MET, resulting in a more mesenchymal phenotype. In addition, epigenetic changes and microRNAs regulate MET, which controls the cellular plasticity of cancer cells [35]. Thus, clinical practice must comprehend how MET and cancer cell plasticity interact. High-plasticity cancer cells are more likely to avoid therapeutic regimens and develop drug resistance. To reverse the mesenchymal phenotype to an epithelial one, targeting MET is being investigated as a potential therapeutic strategy [35]. Targeting several MET-related molecular pathways, including RTKs, TGF-β signaling, and transcription factors connected to the epithelium, has shown encouraging outcomes in several preclinical investigations and ongoing clinical trials. It is essential to comprehend the fundamental mechanisms behind MET dysregulation in cancer cell plasticity while developing cutting-edge therapeutic approaches to thwart tumor development and treatment resistance [36, 37].

### Phenotype switching

The wide range of phenotypic heterogeneity that results from tumor cell plasticity influences the cell’s receptivity to drug treatment and inclination toward metastasis. The cancer stem cell (CSC) theory and the phenotype-switching hypothesis (PSH) both suggest a continuum in which the cellular state is dynamic rather than static. According to their developmental history and the degree of lineage specificity, tumor cells can change their phenotypes, becoming either more or less differentiated or more or less invasive/proliferative (as a result of EMT and MET-like conversions). Switching might happen due to treatment or as an essential feature of tumor development in a three-dimensional environment. An EMT-like subgroup of CSCs resembles invasive, relatively undifferentiated melanoma cells, which are more resistant to therapy [38]. The paradigm of cellular differentiation has undergone a significant shift in recent times. No longer is a cell’s commitment seen as a unidirectional journey. Instead, it is now understood as a highly adaptable state, susceptible to rapid modification in response to environmental cues. This adaptability, known as cell plasticity, has emerged as a critical attribute of cancer biology [39]. The phenomena of plasticity can be governed by both cell-intrinsic factors, such as mutations driving an oncogenic phenotype, and cell-extrinsic factors shaped by the surrounding microenvironment [40].

A prime example of this plasticity is observed in neural crest cells. Renowned for their mobility, these cells embark on migration and undergo EMT, only settling into differentiation once they have navigated to their precise destination within the body. They give rise to a variety of cells within the peripheral nervous system, spanning the spectrum from highly specialized to more generalized types, including Schwann cells, peripheral neurons, osteocytes, chondrocytes, adipocytes, smooth muscle cells, melanocytes, and keratinocytes. Highlighting this intrinsic plasticity, Vidács and colleagues [41], in 2021, observed a remarkable phenomenon in human adult epidermal melanocytes. When cultured in a medium free of cholera toxin and the tumor promoter 12-*O*-tetradecanoylphorbol-13-acetate (TPA), these cells adopted a bipolar and unpigmented morphology [41]. This finding underscores the profound impact of the cellular environment on the state and behavior of cells, further emphasizing the dynamic nature of cellular differentiation and the broader implications it holds for our understanding of complex processes like cancer.

Melanoma plasticity, a dynamic and critical aspect of cancer biology, can be delineated at the transcriptional level based on distinct differentiation markers. Typically, melanoma differentiation stages are bifurcated into two primary transcriptional programs that define proliferative and invasive states [42]. Each of these programs is orchestrated by master regulators that foster the generation of unique transcriptional landscapes. In the context of the “proliferative” phenotype, a well-documented marker is the microphthalmia-associated transcription factor (MITFHigh), coupled with a low expression of AXL (AXLLow). These markers are considered pivotal in signifying the dedifferentiation state. The proliferative phase is characterized by a more differentiated, epithelial-like phenotype. MITF, a key player in melanocyte lineage commitment and pigmentation, activates differentiation genes such as premelanosome protein (PMEL), dopachrome tautomerase (DCT), tyrosinase (TYR), and melan-A (MLANA). Furthermore, the regulation of MITF is influenced by a cadre of upstream activators, including SRY-box transcription factor 10 (SOX10), paired box 3 (PAX3), CAMP-responsive element-binding protein (CREB), and endothelin receptor type B (EDNRB). These factors are generally found at elevated levels within the proliferative phenotype, underscoring their role in this specific transcriptional program [43]. This intricate regulatory network highlights the nuanced control mechanisms governing melanoma plasticity, emphasizing the importance of transcriptional programs in the proliferative phase of melanoma differentiation. Understanding these markers and regulators provides crucial insights into the molecular underpinnings of melanoma and offers potential pathways for therapeutic intervention.

### Role of cancer stem cells

**Fig. 1:**
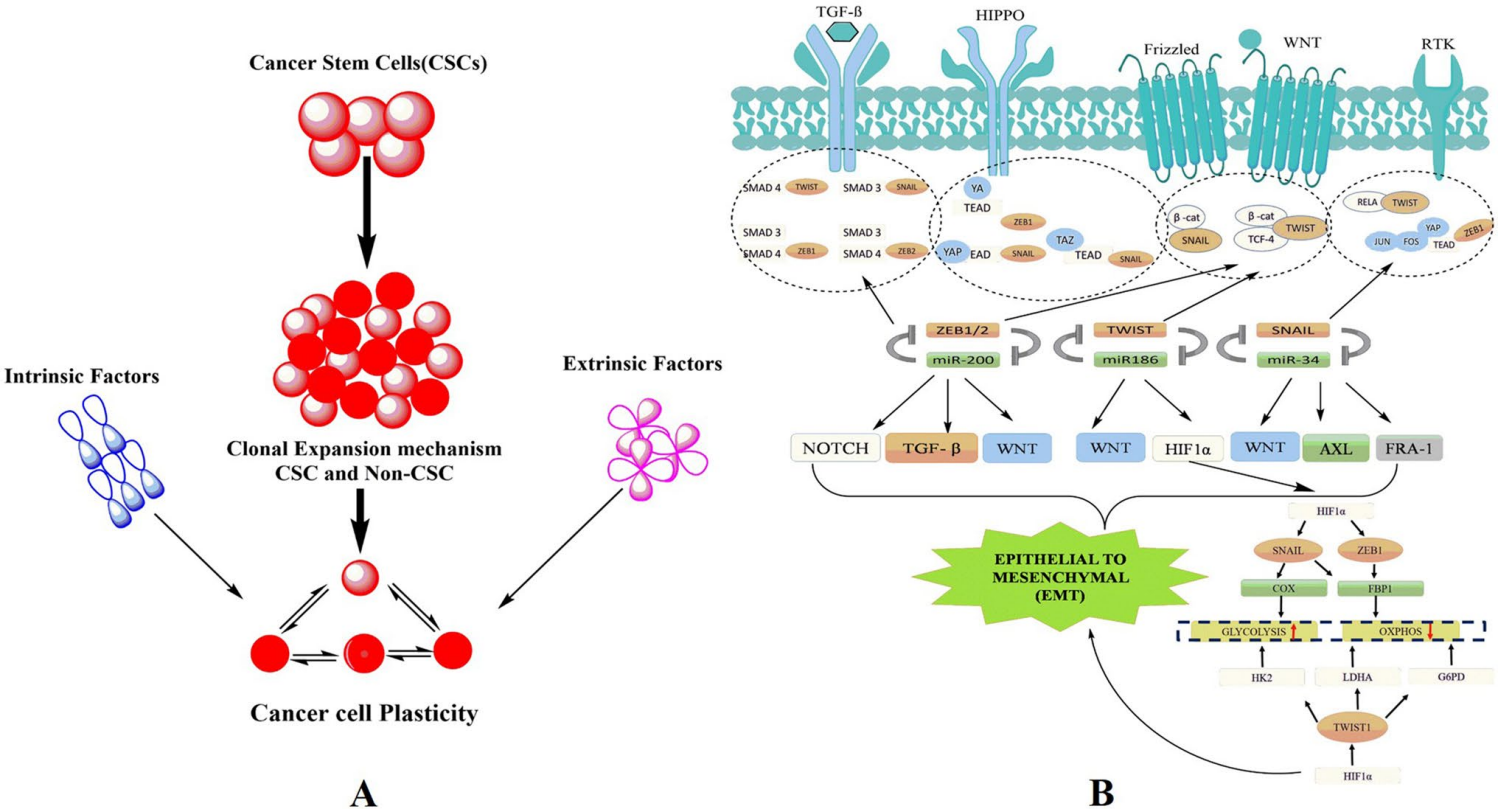
**A** Illustration of the dynamic interconversion between cancer stem cells (CSCs) and non-CSCs. This figure illustrates the minor yet critical subpopulation of tumor mass known as CSCs. It visually elucidates the phenomenon of phenotypic plasticity that empowers both CSCs and non-CSCs to interchange states depending on various intrinsic and extrinsic cellular properties. Intrinsic factors include epigenetic changes that internally modulate cellular activities, while extrinsic factors encompass elements of the tumor microenvironment that externally influence the cells. The figure offers insight into the dynamic nature of cellular identity within tumor masses, emphasizing the impact of diverse cellular and microenvironmental factors on the CSC and non-CSC states. **B** Detailed overview of the regulatory network involving key transcription factors and molecules. This figure comprehensively depicts the transcription factors, including Snail1/Snail2, ZEB1/ZEB2, Twist, and LEF-1, whose expression is intricately modulated by multiple signaling pathways. It outlines the various regulatory molecules that can inhibit the functionality of these transcription factors, thus impacting cellular activities. The figure elaborates on the prevention of LEF-1 activation by GSK-3, which hinders its collaboration with β-catenin and also demonstrates GSK-3’s role in barring the stability and nuclear translocation of Snail1/Snail2. Additionally, the figure highlights the suppression of ZEB1/ZEB2 expression by the miR-200 family of miRNAs and the inhibition of GSK-3 and miR-200 by the kinase Akt, which is activated by most EMT signaling pathways

CSCs represent a unique subset of cells within tumors, characterized by their stem cell–like properties, including self-renewal, differentiation, and the ability to generate a variety of cell types associated with malignant growth [44]. These cells play a critical role in perpetuating cancer by driving tumorigenesis and malignant progression through their inherent mechanisms of self-renewal and differentiation. According to the conventional CSC model, a distinct population of cancerous cells possesses the capability to self-renew, proliferate, and differentiate, thereby mirroring the heterogeneity in morphology and antigen expression observed in the primary tumor (Fig. 1A). This model suggests the existence of a cellular hierarchy within each tumor-specific to an individual patient. Critical to the pathogenesis of these cells is the dysregulation of the self-renewal process. Under normal circumstances, specific genes and signaling pathways tightly regulate self-renewal. However, in CSCs, these regulatory pathways are disrupted, enabling an uncontrolled proliferation of tumor cells without compromising their proliferative capacity [45]. This dysregulation is pivotal in facilitating the sustained growth and resilience of tumors, making CSCs a significant focus for potential therapeutic interventions.

“Tumor stemness” in advanced cancers refers to the ability of tumor cells to self-renew and generate the diverse cellular constituents that compose the tumor mass. This phenomenon takes on added complexity with the involvement of EMT programs, which, beyond inducing mesenchymal traits, are also associated with the expression of stem cell markers and an augmented ability to form mammospheres—a feature indicative of breast epithelial stem cells [46]. The activation of EMT programs is theorized to equip cancer cells with attributes commonly seen in CSCs, such as increased invasiveness and the potential for metastasis. This transformation is particularly significant as EMT-endowed cells can detach from the primary tumor, invade the bloodstream, and navigate to distant sites, a hypothesis supported by the prevalent expression of EMT markers in circulating tumor cells from breast cancer patients [47]. Furthermore, the emergence of cells exhibiting stem cell traits or CSC-like features is not solely dependent on deterministic programs like EMT but can also occur through stochastic cellular transitions. Supporting this, research by Chaffer et al. [48] indicates that a subset of basal-like mammary epithelial cells retains the potential to spontaneously revert to a stem cell state *in vitro*. More notably, these cells can assume CSC-like properties following oncogenic transformation, demonstrating enhanced tumorigenicity and enrichment of CSC markers in xenotransplantation experiments. This dual pathway underscores the plasticity within tumor cell populations and highlights potential therapeutic targets in combating cancer’s adaptability and resilience.

In the dynamic landscape of cancer biology, intriguing transitions have been observed in cultivated breast cancer cell lines. Notably, non-CSCs, isolated through fluorescence-activated cell sorting (FACS), have displayed an unexpectedly rapid regeneration of the CSC population, a phenomenon too prompt to be merely attributed to sorting impurities [49]. These transitions, likely spontaneous rather than induced, hint at the inherent cellular plasticity within a seemingly uniform *in vitro* tissue culture environment. Here, cells unpredictably oscillate with certain regularity among luminal-like, basal-like, and stem-like states. This cellular fluidity was adeptly modeled through Markovian predictions, which successfully anticipated the collective cell-state transition behaviors of FACS-separated luminal, basal, and stem cells [50]. Phillips et al. [51] extended this concept, revealing that even noncancerous breast cells are subject to Markovian cell-state transitions. However, the frequency at which non-CSCs convert to CSCs in breast cancer remains undetermined. The tumor microenvironment (TME) further complicates this scenario. It not only augments classic CSC properties such as self-renewal, differentiation, and sustenance but also promotes metastasis and recurrence. Emerging studies suggest the TME is instrumental in orchestrating the intercellular interactions and signaling mediators that provoke and govern cellular plasticity [52]. Within this niche, an array of cell types, including mesenchymal stem cells (MSCs), cancer-associated fibroblasts (CAFs), immune cells, and exosomes, exert a substantial influence on CSC plasticity. Highlighting the complexity of these interactions, macrophage-derived cytokines, oncostatin M, and osteopontin have been identified as catalysts for the reversion of non-CSCs to CSCs while enhancing the colony-forming prowess of CSCs. Furthermore, the plasticity in various cancers is modulated through an intricate network of signaling pathways such as Notch, IGF-II/IGF1R, c-Met/FRA1/HEY1, and FAK, primarily driven by fibroblasts and CAFs originating from MSCs [53]. These findings underscore the multifaceted nature of CSCs and the surrounding microenvironment, prompting a re-evaluation of therapeutic strategies targeting these elusive cells.

Beyond the direct interactions between cancer and stromal cells, the TME orchestrates a complex milieu through substances secreted by various cell types, establishing sophisticated networks of interlinked cells [54–57]. Growing research underscores how this intricate interplay can significantly mold cancer cells’ stem-like characteristics and phenotypic agility. Within this environment, CAFs emerge as pivotal contributors to multiple facets of tumor progression [58]. Diverse studies highlight the profound impact of CAFs on CSC plasticity across various cancers. For instance, c-Met/FRA1/HEY1 signaling was implicated in modulating CSC plasticity in hepatocellular carcinoma, FAK signaling in pancreatic adenocarcinoma, and IGF-II/IGF1R signaling pathway in lung cancer [59]. Moreover, a study by Du et al. [60] revealed that the intensity of Notch1 signaling within dermal fibroblasts, originating from mesenchymal stem cells, directly correlated with their ability to dictate melanoma’s aggressiveness, stemness, and phenotypic flexibility. The immune system, another integral component of the TME, exerts a considerable influence over CSC plasticity. For instance, in the wake of chemotherapy, macrophages have been known to secrete oncostatin M (OSM), a cytokine from the IL-6 family, propelling the dedifferentiation of aggressive stem cells into triple-negative breast cancer cells [61]. This process, as described by Junk et al. [62], might be facilitated by synergistic STAT3/SMAD3 signaling. Furthermore, cancer-associated adipocytes can also exude OSM, potentially fostering stemness [63]. Recent insights from studies examining EMT in breast, pancreatic, and ovarian cancers suggest that the transition from epithelial-like to mesenchymal-like cells is not an absolute switch but rather a gradual dimming process. This implies that the phenotypic and functional shifts in many malignancies navigate along a spectrum. The term “partial EMT,” or pEMT, has been coined to denote this nuanced, incomplete progression toward a mesenchymal state [64]. This revelation not only enhances our understanding of tumor biology but also signifies the necessity for more targeted therapeutic strategies that consider the fluidity of cancer cell states.

## Molecular mechanisms of cancer cell plasticity

In the intricate landscape of oncology, the molecular mechanisms underlying cancer cell plasticity represent a pivotal point of study. Delving into this realm, this section explicates the dynamism of cancer cells at the molecular level, an attribute that not only confers upon them the notorious ability for therapeutic evasion but also underpins their aggressive metastatic potential. This comprehensive analysis lays the groundwork for understanding the multifaceted nature of cancer cells, setting the stage for the subsequent sections that further dissect the implications of cellular plasticity. As we transition from the molecular constituents to their real-world ramifications, it becomes increasingly clear that cancer cell plasticity is not an isolated phenomenon but a critical cog in the complex machinery of cancer progression and treatment resistance. Thus, the insights garnered in this section are instrumental in prefacing the upcoming discussions on therapeutic strategies and clinical challenges, ensuring a holistic comprehension of the disease’s intricacies.

### Signaling pathways involved in EMT and MET

Embryonic development and tissue repair processes hinge on a critical biological phenomenon known as the EMT. Beyond its physiological roles, EMT is also implicated in pathological conditions such as cancer progression and organ fibrosis. Initiation of EMT is characterized by the upregulation of specific transcription factors that alter gene expression, culminating in the loss of cell–cell adhesion, cytoskeletal reorganization, and a comprehensive shift from an epithelial to a mesenchymal phenotype. A parallel process observed in vascular endothelial cells is termed the endothelial-mesenchymal transition (EndMT). Several key signaling cascades, including those mediated by TGF-β, bone morphogenetic protein (BMP), Wnt-β-catenin, Notch, Hedgehog, and receptor tyrosine kinases, are instrumental in driving the transcriptional reprogramming inherent in EMT. These pathways do not operate in isolation; they are activated in response to a confluence of microenvironmental cues such as hypoxia, interactions with the ECM, and the presence of growth factors and cytokines. This section delves into the intricate interplay between these signaling pathways and their responsiveness to the microenvironment. It further explores how such interactions influence the expression and activity of transcription factors pivotal in EMT induction and, by extension, tumorigenesis. By unraveling these complex pathways, we open avenues for the therapeutic manipulation of EMT. This understanding holds the promise of harnessing EMT for tissue regeneration, fibrosis resolution, and arresting cancer metastasis, presenting a multifaceted approach to disease management and treatment [65].

#### EMT/MET-inducing transcription factors

The EMT is orchestrated by an array of transcription factors that precipitate profound alterations in cellular physiology. Throughout the EMT process—and its reverse, the MET—cells undergo dramatic changes: intercellular adhesions dissolve, epithelial polarity diminishes, the cytoskeleton undergoes dynamic restructuring, and basement membranes disassemble, among other transformations. At the heart of these coordinated physiological shifts are specific transcription factors known as EMT-TFs. These regulatory proteins oversee the intricate processes of EMT, with some even transitioning from acting as transcriptional repressors to activators upon interaction with certain signal transduction pathway effectors. Conversely, another group of transcription factors, termed MET-TFs, plays a pivotal role in maintaining the delicate EMT/MET equilibrium. These factors bolster epithelial attributes in both normal and neoplastic cells, chiefly by repressing the transcription of mesenchymal markers and establishing reciprocal inhibitory loops with EMT-TFs [66–69]. In addition to their regulatory roles, several MET-TFs, including CDH1, ZO-1, and those encoding claudin-4 and claudin-5 (CLDN4 and CLDN5), directly stimulate the transcription of genes that codify proteins essential for epithelial lineage specification [70, 71].

These orchestrated molecular events underscore the complexity of cellular transitions during EMT and MET, highlighting the necessity for a nuanced understanding of these processes in both health and disease. Transcription factors such as Snail1 and Snail2 (also recognized as Slug), along with various basic helix-loop-helix (bHLH) factors like ZEB1 and ZEB2, play pivotal roles in the orchestration of EMT. Twist, another significant transcription factor [72, 73], part of the T cell factor (TCF) family, can be directly spurred by lymphoid enhancer binding factor 1 (LEF-1) [74].

These specific proteins function by binding to promoter regions of genes, particularly those associated with cell–cell adhesion, effectively repressing their expression. This transcriptional regulation marks a critical initiation step in the progression of EMT.

Central to the tight regulation of EMT are the Snail family of transcriptional repressors. Snail1 and Snail2 exert their influence by binding to the CDH1 promoter, inhibiting the translation of CDH1, the gene responsible for E-cadherin production [75, 76]. An accumulation of Snail1 in the nucleus, coupled with diminished E-cadherin levels, has been associated with breast cancer phenotypes prone to metastasis [77]. Interestingly, circulating tumor cells from patients with metastatic hepatocellular carcinoma (HCC) exhibited Snail1 levels up to 20 times higher than those from patients with nonmetastatic HCC [77].

Snail2, meanwhile, is implicated in various developmental processes such as gastrulation, neural crest formation and migration, and the initiation of EMT in cancer metastasis. The overexpression of either Snail1 or Snail2, which often results in the induction of EMT, hints at the reactivation of developmental programming within metastatic carcinomas, albeit in ways that vary depending on the physiological context within the organism. This overexpression is closely associated with increased tumor metastasis, underlining the significant role of these transcription factors in cancer progression.

Twist1 and Twist2, integral members of the bHLH transcription family, are fundamental to cancer proliferation [78]. Specifically, Twist1 has been observed to engage with the SNAI2 promoter in human mammary cells, elevating gene expression to instigate EMT. This mechanism correlates with findings that show a pronounced prevalence of Twist1 in metastatic mammary tumors compared to less metastatic variants [78, 79]. Intriguingly, Twist1 manifests more predominantly in mouse models of atypical ductal hyperplasia, an initial phase in primary breast tumor evolution (Fig. 1B) [80].

Attention has also converged on the ZEB family of transcriptional repressors, notable for their regulatory role in cancer progression and critical function in neural crest development. ZEB1 and ZEB2 interact with the bipartite E-box segments of DNA flanking the CDH1 gene, inhibiting its promoter activity. Furthermore, they enhance the transcription of genes encoding matrix metalloproteinases (MMPs), thereby establishing a connection between ZEB1 and ZEB2 and various matrix remodeling pathways related to EMT. These remodeling processes may subsequently initiate the transmission of additional extracellular signals. The regulatory complexity extends to involve microRNAs; specifically, five microRNAs (miRNAs) within the miR-200 family, possessing identical targeting sequences, act to diminish ZEB protein concentrations at the CDH1 promoter. In a reciprocal interaction, ZEB1 and ZEB2 bind to the miR-200 E-box promoters, creating a feedback loop instrumental in the modulation of EMT [81]. This intricate transcriptional control network underscores the multifaceted regulatory mechanisms governing EMT and its pivotal role in cancer progression.

#### TGF-β and BMP signaling in EMT/MET

TGF-β signaling stands out as the foremost pathway in triggering EMT, operating through a spectrum of intracellular intermediaries depicted in Fig. 3. The extensive TGF-β superfamily encompasses various ligands, notably three TGF isoforms (TGF-1, TGF-2, and TGF-3) and six BMP isoforms (BMP2 to BMP7), each playing distinct roles in signaling activation. For instance, TGF-1 is pivotal in governing EMT across numerous systems, notably in cancer and fibrosis scenarios [82]. In contrast, TGF-2 primarily modulates EMT throughout cardiac development [83], and TGF-3 is instrumental during palate formation [74]. EMT induction is attributed to BMP2 and BMP4 in the realm of cancer, with BMP4 extensively engaged across diverse tissues. Its role in rekindling developmental pathways is evident from its marked presence in invasive epithelial tissues relative to normal colonic mucosa [84, 85], as well as in contexts of breast cancer [86], and fibrosis [87]. Intriguingly, BMP7 emerges as a consistent inhibitor of EMT, often promoting an epithelial cell phenotype, while BMP5 counteracts TGF-induced EMT. These instances underscore the specific influences of individual BMP isoforms on disease progression and manifestation [88]. TGF-β signaling intricacies extend to receptor involvement, where type I and type II TGF receptors (TGF-RI and TGF-RII) converge to establish a heterotetrameric receptor complex. The receptor landscape in mammals is diverse, featuring five type II and seven type I receptors [89–91]. The dynamics of ligand-receptor interactions further evolve in epithelial and endothelial cells, where TGF type III receptors and auxiliary proteins like glycan, endoglin, and cripto potentially alter ligand binding affinities at the cellular membrane [92, 93]. Notably, BMP signaling diverges from TGF-β pathways, employing a distinct type II BMP receptor in place of TGF-RII [94, 95]. This nuanced interplay of signaling pathways, receptor types, and ligand-receptor dynamics illustrates the complexity underlying EMT induction and regulation, emphasizing the significance of each component in the broader context of developmental and pathological processes.

#### SMAD-dependent signaling

**Fig. 2:**
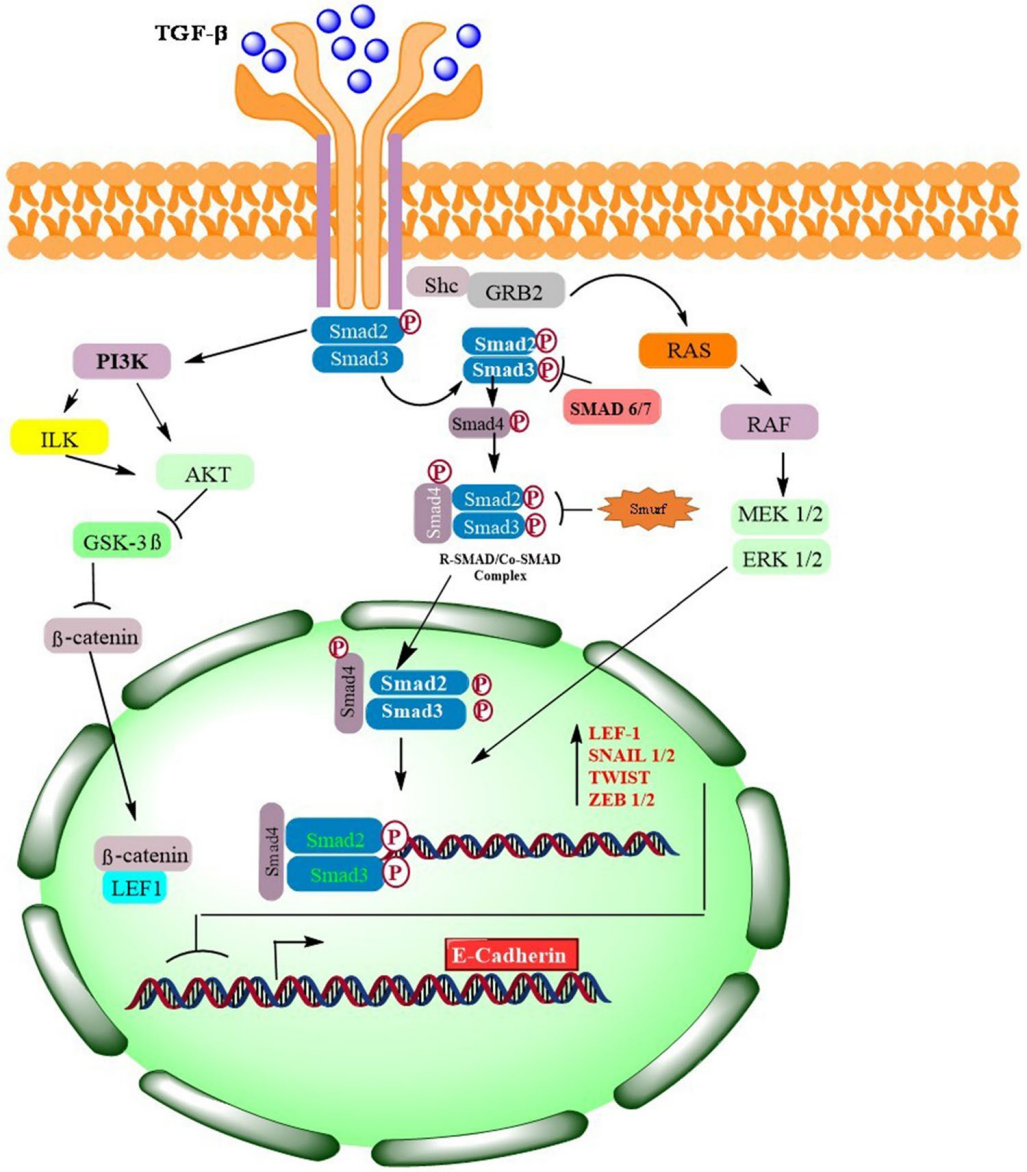
Detailed representation of TGF receptor–mediated signaling pathways and their regulatory mechanisms. This figure provides a thorough visual exploration of the complex interactions and activities instigated by the binding of TGF ligands to their type II and type III receptors (TGF-RII and TGF-RIII). It illustrates the consequential recruitment and phosphorylation of the type I receptor (TGF-RI), a pivotal action that triggers multiple signaling pathways. This intricate signaling network, including pathways controlled by SMAD2/SMAD3, Ras, and PI3K, is detailed in the figure, emphasizing their crucial role in activating specific transcription factors. The figure elaborates on the cascade effect that ensues, leading to the expression of genes that encode transcription factors instrumental in initiating epithelial to epithelial-to-mesenchymal transition (EMT). Furthermore, the figure delineates the activation of Akt by SMAD-independent pathways, such as PI3K and ILK. Akt’s subsequent limitation of GSK-3β activity is visually explained. This limitation is significant as GSK-3β is a kinase that inhibits the nuclear translocation of Snail and β-catenin, critical components in cellular transformation and movement. Moreover, Fig. 3 highlights the role of Smurf2 and SMAD6/SMAD7 in inhibiting SMAD signaling. It explains Smurf2’s function in degrading the active complex SMAD2/SMAD3/SMAD4 and SMAD6/SMAD7’s blockage of SMAD2/SMAD3 binding and phosphorylation at TGF-Rs

TGF-RII’s Ser/Thr kinase activity catalyzes the phosphorylation of TGF-RI, setting the stage for the recruitment of transcription factors SMAD2 and SMAD3—also known as mothers against decapentaplegic homologs 2 and 3 (SMAD2/SMAD3). This interaction occurs in the Gly/Ser (GS)-rich domain of TGF-RI, as depicted in Fig. 2. Subsequently, the Ser residues in the C-terminal domain of these SMADs undergo phosphorylation, facilitating the formation of a complex with the coactivator SMAD4. Crucially, the phosphorylation of MH2 domains by TGF-R1 not only primes the oligomerization of SMAD2/SMAD3 with SMAD4 but also unveils the nuclear localization signal, a critical component for the nuclear import of the R-SMAD/SMAD4 complex. This signal engages importins b1, 7, and 8, orchestrating the complex’s translocation into the nucleus [96–98].

**Fig. 3:**
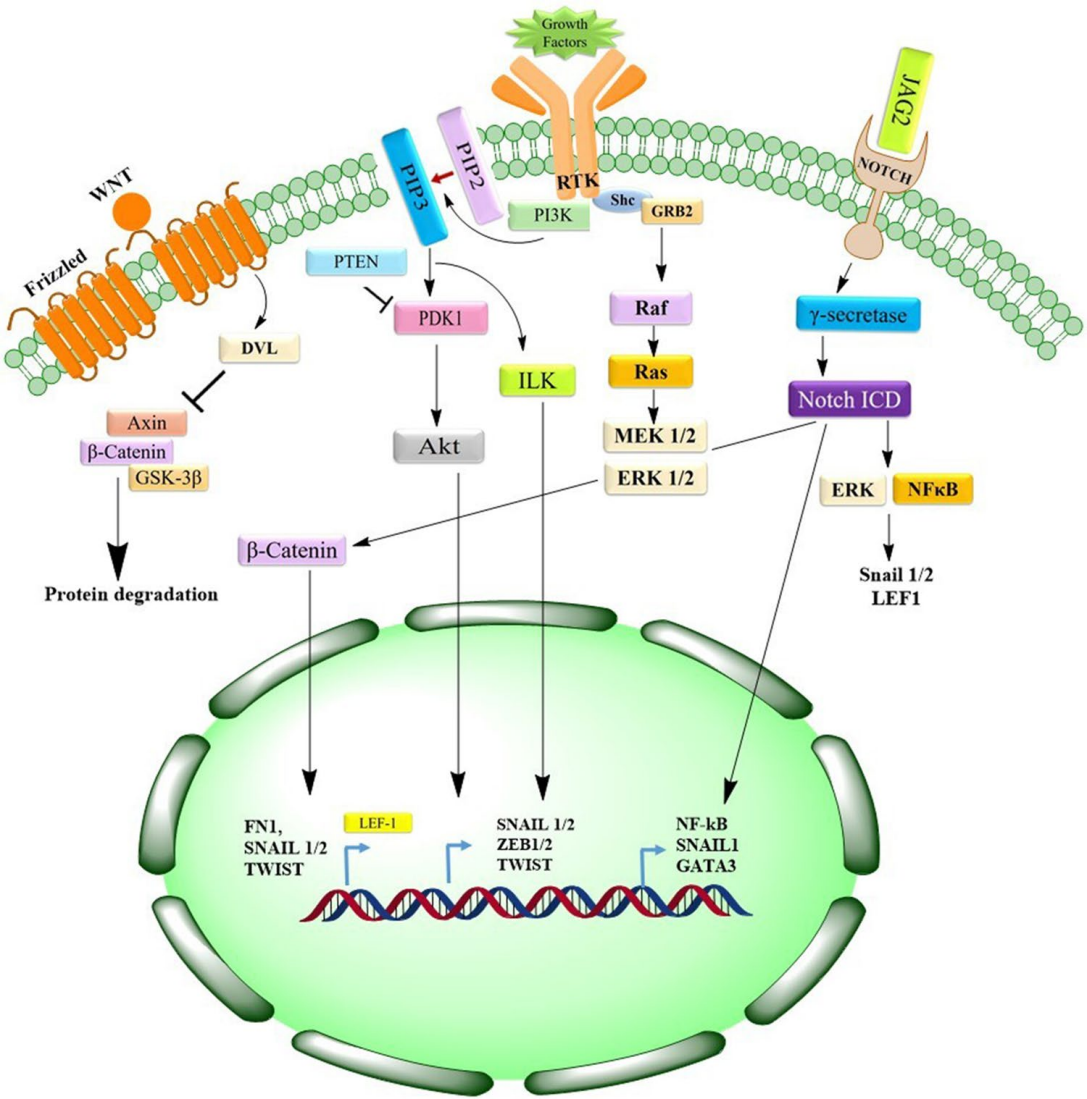
Detailed mechanism of EMT-related gene activation via various pathways. This figure describes the mechanisms that underlie the Dvl-dependent regulation of GSK-3β, a key kinase involved in the breakdown of cytoplasmic β-catenin. The illustration outlines the sequence initiated by the binding of Wnt ligands to Frizzled receptors. This binding and activation event facilitates the nuclear localization and accumulation of β-catenin, subsequently activating the LEF-1 transcription factor. The figure further emphasizes the consequential stimulation of the production of several EMT-related genes, which are critical in tumor progression and metastasis. The processes leading to the Notch intracellular domain (ICD) release by the γ-secretase enzyme in response to the interaction between JAG2 and its receptor Notch are also depicted. The figure highlights the roles of various pathways, including ERK and NF-κB, activated by the Notch ICD, in the induction of the Snail1/Snail2 and LEF-1 transcription factors

Upon arrival in the nucleus, SMAD complexes target regulatory regions, initiating the transcription of key genes associated with EMT. Notably, R-SMAD complexes can ally with Snail1, suppressing the transcription of genes that encode E-cadherin and occludin. Moreover, they can directly bind to the SNAI1 promoter, stimulating its transcription [99]. The sphere of influence of R-SMADs extends to the ZEB transcription factors and the high mobility group factor HGMA2. These elements, vital in modulating the expression of SNAI1, SNAI2, and Twist, are directly affected by R-SMAD binding, underscoring the multifaceted role of SMAD complexes in the intricate network of EMT regulation [100].

#### SMAD-independent signaling

TGF-receptor complexes orchestrate numerous SMAD-independent pathways, commonly intersecting with RTK signaling and SMAD protein functions. Notably, the PI3K-Akt pathway holds a multifunctional role in steering EMT processes. TGF can either directly instigate PI3K activity or proceed indirectly through the transactivation of EGF and PDGF receptors [101, 102].

Research has documented the ability of TGF-β to trigger PI3K and Akt pathways across various cell types [101, 103]. Once activated, PI3K catalyzes the transformation of phosphatidylinositol 4,5-bisphosphate (PIP2) into phosphatidylinositol 3,4,5-trisphosphate (PIP3), a phospholipid membrane constituent that recruits Akt. Subsequently, Akt undergoes phosphorylation by phosphoinositide-dependent kinase 1 (PDK1) [104]. The PI3K catalytic subunit, p110, is prone to mutations in cancer scenarios, often leading to escalated PIP3 production and aberrant Akt stimulation. Moreover, integrin activation can prompt integrin-linked kinase (ILK) to phosphorylate Akt [105]. Within mammalian cells, three Akt isoforms exist. When Akt2 is activated, it phosphorylates heterogeneous nuclear ribonucleoprotein E1 (hnRNPE1), which normally binds to the 3′ untranslated region (UTR) of messenger RNAs (mRNAs), inhibiting translation. Phosphorylation displaces hnRNPE1 from mRNAs coding for disabled homolog 2 and the interleukin-like EMT inducer, thus promoting the synthesis of proteins that drive EMT transcription factor expression. Furthermore, in squamous cell carcinoma cells, Akt fosters EMT by energizing nuclear factor kappa B (NF-κB), amplifying SNAI1 transcription [106].

#### Wnt signaling

Wnt signals are conveyed across the plasma membrane through the collaboration of Frizzled and low-density lipoprotein receptor–related protein (LRP) receptors. In a signal-absent environment, a complex comprising GSK-3β, axin, and the tumor suppressor adenomatous polyposis coli (APC) phosphorylates β-catenin. This action retains it within the cytoplasm and earmarks it for proteasomal degradation [107]. The signaling paradigm shifts when Wnt ligands engage Frizzled, leading to the phosphorylation of LRP6 by GSK-3β and the subsequent recruitment of Disheveled (Dvl) and axin to the plasma membrane. This repositioning hinders GSK-3β’s access to axin, thereby thwarting β-catenin phosphorylation and paving its path to the nucleus. Upon Wnt-β-catenin pathway activation, GSK-3β confinement within the cytoplasm stabilizes Snail1. In the nucleus, β-catenin allies with TCF/LEF family transcription factors to promote EMT. Illustrating its role during gastrulation, β-catenin interacts with CDH1, impeding its transcription through a complex formed with LEF-1 [108]. Concurrently, the Wnt-GSK-3β-β-TRCP1 (β-transducing repeat-containing protein 1) axis propels Snail2 activation, fostering EMT and repressing breast cancer 1 (BRCA1; early onset) expression by latching onto its promoter and recruiting a histone demethylase. The correlation between BRCA1 loss and aggressive basal-like breast cancer underscores the significance of this mechanism [109]. Further evidence of Wnt’s role in EMT induction comes from observed decreases in E-cadherin and increases in fibronectin consequent to the accumulation of β-catenin in the nucleus, suggesting Wnt’s influence through Snail2 [110]. Moreover, Wnt signaling has been associated with heightened Twist expression in mammary epithelial cells, reinforcing its integral role in modulating cellular transitions.

#### Notch signaling

The Notch receptor, structured with an intracellular domain harboring a nuclear localization motif (Notch intracellular domain (NICD)) and an extracellular domain, undergoes cleavage by γ-secretase and TACE, liberating the NICD. This cleavage facilitates NICD’s translocation to the nucleus when proximal Notch signaling is initiated [111, 112]. Within the nucleus, NICD engages with DNA-bound CSL transcription repressor complexes (CBF1, Su(H), LAG1), stimulating genes pivotal for tumor proliferation, including NF-κB, Akt, and p21 [112–114]. This pathway not only directly influences SNAI1 expression through upregulation of hypoxia-inducible factor 1 (HIF-1) but also indirectly governs EMT via several signaling cascades, encompassing NF-κB and β-catenin, along with a spectrum of regulatory miRNAs. Notch’s role extends to the vascular framework, where its overexpression in endothelial cells precipitates the loss of vascular endothelial (VE) cadherin, instigating EndMT. In the context of lung cancer, curtailing Notch1 appears to mitigate invasive propensities and partially reverse EMT. The intricacy of Notch signaling further unfolds in its regulation of EMT through the miR-200 family, modulated by the induction of GAT-binding protein 3 upon the binding of the Notch ligand Jagged2 (JAG2). This interaction suppresses miR-200, thereby favoring EMT [115]. Concurrent research indicates that miR-200 counteractively targets JAG1, establishing a feedback loop that governs Notch activation, as depicted in Fig. 3 [115]. This complex interplay underscores the multifaceted nature of Notch signaling in both the direct and indirect orchestration of EMT.

### Role of microRNAs and long noncoding RNAs

Noncoding RNAs (ncRNAs) represent a class of molecules characterized by their inability or limited potential to encode proteins. Intriguingly, the majority of human genes yield RNAs that serve functions beyond the scope of protein translation [116]. Recent strides in high-throughput technologies coupled with multidisciplinary approaches have illuminated the complex signaling tapestry woven by ncRNAs within human cellular machinery. Among these, long noncoding RNAs (lncRNAs) and miRNAs stand out as principal subclasses of non-protein coding transcripts. These ncRNAs orchestrate a wide array of biological narratives, extending their influence to the nuanced modulation of gene expression. Their roles are diverse, underscoring the intricate layers of regulatory control they contribute to the broader genomic dialog [116]. This revelation not only expands our understanding of genetic regulation but also opens new avenues for exploring the molecular underpinnings of various biological processes.

#### miRNAs regulate cancer cell plasticity and tumor progression

miRNAs are succinct, noncoding RNA fragments, typically 19–23 nucleotides in length, that execute a critical regulatory function in the post-transcriptional governance of gene expression. They achieve this by selectively binding to complementary sequences within target mRNAs, an interaction that either impedes translation efficiency or prompts mRNA destabilization. Within the cellular milieu, miRNAs are pivotal arbiters in various processes, including cell division, proliferation, programmed cell death, or apoptosis. Emerging research underscores a profound association between miRNA dysregulation and various malignancies [117, 118]. Depending on their specific mRNA targets, miRNAs can assume a dualistic nature, acting as either tumor suppressors or oncogenic promoters. The nuanced modulation of miRNA expression offers insights into malignant cells’ developmental lineage and maturation stage. Moreover, distinctive miRNA profiles serve as reliable molecular signposts for classifying poorly differentiated tumors. In breast cancer research, a conspicuous disparity in miRNA expression has been observed between cancerous cells and their normal counterparts [118]. Specific miRNAs, such as let-7e, miR-151-5p, miR-222, miR-21, miR-155, and miR-221, manifest elevated levels in malignant tissues, as identified through comprehensive miRNA expression profiling studies [119–121].

This elevation not only underscores the potential role of miRNAs in cancer diagnostics but also suggests the feasibility of differentiating malignant from normal tissues based on miRNA signatures.

Numerous studies indicate that cancer cells often undergo EMT in reaction to TGF-β stimulation. TGF-β orchestrates EMT in advanced cancers by directly enhancing the transcription factors ZEB, Snail, and Twist. Research has pinpointed several microRNAs, including miR-200, miR-21, and miR-31, as potential mediators in the TGF-β-induced EMT pathway. Notably, TGF-β stimulation has markedly increased the expression of miR-21 and miR-31. These microRNAs, targeting TIAM1—a guanine exchange factor for Rac GTPase—synergize with TGF-β to facilitate EMT [122].

Within the miR-200 family, which includes miR-200a, miR-200b, miR-200c, miR-141, and miR-429, there is a growing consensus that these microRNAs exert significant control over EMT, a process instrumental in tumor metastasis. An intriguing observation is the substantial reduction in all five miR-200 family members in cells subjected to TGF-β-induced EMT, as well as in invasive breast cancer cell lines reliant on SMAD signaling. Central to the action of miR-200 are ZEB1 and SIP1, known repressors of E-cadherin transcription. By directly inhibiting the mRNA of ZEB1 and SIP1, miR-200 maintains E-cadherin expression and epithelial morphology. Adding a layer of complexity, ZEB1 and SIP1 reciprocally regulate miR-200 by binding to a conserved sequence in the miR-200 promoter region, functioning as its repressors in mesenchymal cells. This establishes a feedback loop that reinforces the mutual regulation between miR-200 and ZEB1/SIP1, a dynamic that underscores the intricate molecular interplay governing EMT and potentially offering therapeutic avenues [123].

#### lncRNAs in regulating cancer cell plasticity

The advent of sophisticated technologies like high-resolution microarrays and genome-wide sequencing has led to identifying numerous unique ncRNA transcripts. Astonishingly, active transcription is estimated to encompass approximately 70% of the genome [124]. Among these, lncRNAs, ranging from 200 bp to 100 kb pairs in length, constitute a substantial fraction of the ncRNA repertoire. Despite numbering in the hundreds, lncRNAs remain the most enigmatic, with a vast majority yet to be functionally characterized [125].

However, recent studies are progressively uncovering the significant roles lncRNAs play in various cancers, including but not limited to breast, prostate, pancreatic, and hepatocellular carcinomas. These molecules can wield tumor-suppressive or oncogenic effects, thereby influencing the pathogenesis and progression of these malignancies. As research continues, the intricate roles of lncRNAs and other ncRNAs are anticipated to become more defined, potentially paving the way for novel therapeutic strategies in cancer treatment.

One prominent lncRNA, HOTAIR (HOX antisense intergenic RNA), spans 2.2 kb and resides on chromosome 12q13.13. HOTAIR levels are aberrantly high in various prevalent human cancers, and a wealth of evidence implicates it in drug resistance, metastasis, and the initiation of cancer. Its expression serves as a potent prognostic indicator for several cancers. For instance, Li et al. [126] determined that high HOTAIR levels in laryngeal squamous cell carcinomas foster PTEN methylation, thereby driving carcinogenesis. Notably, elevated HOTAIR expression has been identified in primary breast tumors and their metastases, and its presence in primary tumors is a robust predictor of subsequent aggressive disease. Conversely, reducing HOTAIR expression appears to curtail cancer development and tumor growth.

Another lncRNA, ANRIL (antisense noncoding RNA at the INK4 locus), has been found to play a pivotal role in cancer progression. Functionally akin to HOTAIR, ANRIL suppresses the activity of the p15 INK4B gene, a crucial tumor suppressor involved in cell cycle regulation, senescence, and stress-mediated apoptosis [127]. This suppression occurs as ANRIL binds to and recruits PCR2, a histone-modifying complex. In a groundbreaking discovery, Nie et al. [128] uncovered an additional mechanism by which ANRIL downregulates the transcription of KLF2 and P21, two genes integral to cellular regulation. These insights suggest that lncRNAs like HOTAIR and ANRIL might promote carcinogenesis by strategically silencing tumor suppressor genes. The nuanced roles of such lncRNAs in cancer underscore their potential as targets for innovative therapeutic interventions.

The intricate roles of miRNA and lncRNA in orchestrating cancer characteristics have garnered substantial attention, acknowledging their critical functions as cancer regulators. Emerging studies are increasingly unraveling the complex interconnections between miRNA and lncRNA. These multifaceted molecules can act both as destabilizers and as decoys in cellular pathways. For instance, the notable lncRNA HOTAIR has been identified as a key player in perpetuating the EMT process and sustaining the CSC population in breast cancer by modulating the expression of HoxD10, which, in turn, maintains the levels of pivotal genes like C-myc, Twist, and miR-9 [129]. In a contrasting mechanism, miR-34a has been found to bind directly to a region of HOTAIR mRNA, inhibiting its expression in prostate cancer cells [130].

This underscores the undeniable presence of an extensive ncRNA network that meticulously regulates EMT-associated genes and relevant signaling cascades, thereby directing cancer cell adaptability and the CSC phenotype. Given the evident significance of miRNA and lncRNA interplay in defining cancer and CSC properties, there is a compelling prospect for therapeutic interventions targeting these and associated oncogenes and signaling pathways. Nonetheless, the vast multitude of ncRNAs and the intricacies of their interactions continue to pose a challenge, leaving their diverse physiological and pathological roles largely uncharted scientific territory.

#### Chimeric RNA and cancer cell plasticity

The landscape of cancer genetics is often altered by somatic variations, particularly gene fusions, leading to the emergence of chimeric RNAs. These hybrid molecules, resulting from the fusion of exons from different genes, contribute significantly to the phenotypic heterogeneity observed within cancer cell populations by introducing novel functionalities not found in normal cells. These functionalities have profound implications for various cancer-related pathways, impacting oncogenesis, impeding programmed cell death, and notably enhancing cancer cell plasticity. The consistent recurrence of certain chimeric RNAs across various cancer types, along with their established roles in oncogenic pathways, positions them as potent biomarkers for cancer diagnosis. They are not mere molecular anomalies but active participants in the cancer narrative, often conferring survival advantages to cancer cells, including resistance to conventional drug therapies. This resilience against treatment underscores the potential of chimeric RNAs as critical targets for developing tailored therapeutic strategies in precision medicine. Recent research underscores the adaptive advantage conferred by chimeric RNAs, revealing their capacity to endow cancer cells with novel properties that facilitate drug resistance. This revelation is particularly crucial in the context of personalized cancer treatment, as targeting these chimeric entities could disrupt the survival mechanisms of cancer cells, paving the way for more effective and individualized therapeutic regimens. Understanding chimeric RNAs from an evolutionary standpoint is pivotal in demystifying the complex progression of cancer. By studying how these unique RNA molecules influence cancer cell evolution, we gain invaluable insights into the dynamic interplay of genetic factors that drive malignancy. This knowledge not only illuminates the molecular underpinnings of cancer but also guides the rational design of next-generation drugs. These advanced therapeutic agents, tailored to the genetic makeup of each patient’s cancer, hold the promise of improved outcomes, heralding a new era of personalized oncology [131].

## Unraveling the genetic mechanisms behind cancer cell plasticity

Cancer embodies not just a static condition but an evolutionary malady, characterized by continuous cellular adaptations and modifications in response to the burgeoning demands of proliferation. This relentless evolution engenders a mosaic of cancer cell subpopulations, each exhibiting distinct morphological attributes and a spectrum of tumorigenic potentials and functionalities. Throughout this dynamic journey—termed cancer cell plasticity—cancer cells oscillate among diverse phenotypic states. “Cellular plasticity” refers to the inherent ability of cells to transition between these phenotypes, a transformative process driven by both genetic and epigenetic shifts in response to environmental cues. This adaptability is not only central to normal biological processes like growth and tissue repair but is also vividly illustrated during embryonic development when the inner cell mass of the blastocyst is primed to diverge into one of three germ layers: endoderm, mesoderm, or ectoderm. This pivotal decision, guided by morphogens and external signals, dictates the fate of embryonic stem cells (ESCs) into specific lineages [132, 133]. It is crucial to recognize that this lineage commitment reflects a period of heightened cellular flexibility during early development, which subsequently diminishes as cells mature and specialize. Advances in technology, including single-cell RNA sequencing and advanced computational analytics, have brought the intricate details of cancer into sharper focus. These innovations enable the profiling of cancer landscapes with unprecedented precision, thereby illuminating the spatial and temporal dynamics of cancer cell reprogramming. This review delves into the quintessential features of cancer plasticity, such as the EMT and the concept of cancer stemness. It also explores the confluence of intrinsic genetic elements (e.g., epigenetic modifications, variances in DNA, RNA, or protein compositions) and extrinsic environmental determinants (e.g., tissue architecture, extracellular conditions) that culminate in this plasticity. The resultant tumor heterogeneity, a byproduct of cancer plasticity, poses formidable challenges to treatment strategies by fostering therapeutic evasion and drug resistance. Thus, decoding the cellular and molecular orchestrations that underlie this plasticity becomes a task of paramount importance [134].

The ensuing sections will dissect the primary contributors to cancer plasticity, categorizing them into cell-intrinsic components like transcription factors and epigenetic modifications, cell-extrinsic elements encompassing stromal cells, microenvironmental signals, and more. Each plays a critical role in the tapestry of cancer’s evolutionary journey, influencing its trajectory and therapeutic outcomes.

### Intrinsic factors

#### Genetic mutations

Cancer fundamentally anchors itself in the realm of genetics. As Vogelstein and Kinzler [135] precisely articulated, “Cancer is, in essence, a genetic disease.” This concept hinges on the understanding that cancer’s insidious progression is fueled by an accumulation of genetic disruptions—mutations, chromosomal rearrangements, and various aberrations—that evolve over time. The past decades have witnessed an intensified focus on dissecting these genetic perturbations, a pursuit that has been significantly propelled by advancements in DNA sequencing technologies [136, 137]. Central to the genesis of many cancers are mutations in pivotal genes, notably oncogenes and tumor suppressor genes. These genetic deviations not only instigate the initial transformation of normal cells but also foster intratumoral heterogeneity, a breeding ground for various cancer subclones. Such genetic diversity within tumors can arise from random mutations accumulating over time, environmental assaults, or therapeutic interventions [138]. Characteristically, cancer cells exhibit an array of chromosomal instabilities, including aneuploidy, as well as structural rearrangements like translocations, deletions, or amplifications [139]. These large-scale genomic alterations coexist with more subtle changes, such as localized point mutations, which can significantly recalibrate cellular characteristics. These genetic modifications can catalyze either gain-of-function phenotypes, amplifying traits associated with “stemness,” or loss-of-function phenotypes, disrupting genes regulating the cell cycle. Either scenario can profoundly alter the cellular behavior within the cancer milieu.

Moreover, defects in DNA replication and repair mechanisms can directly impinge on genes dictating cellular differentiation or stemness, thereby driving cancer cell plasticity. Emblematic of this are hematological malignancies like chronic myeloid leukemia (CML) and acute lymphoid leukemia (ALL), where chromosomal translocations in hematopoietic stem cells trigger leukemogenesis. One archetypal example is the reciprocal chromosomal translocation t(9;22), culminating in the notorious BCR-ABL fusion protein, a molecular aberration known for its role in deregulating the cell cycle. In acute myeloid leukemia (AML), mutations in cardinal genes such as DNMT3A, NRAS, and NPM1, especially in the context of specific translocations like t(8;21) or t(15;17), are implicated in disease onset. In solid tumors like colon cancer, genetic anomalies can have far-reaching effects. For instance, the loss of APC disrupts the Wnt signaling pathway in cancer stem cells, hyperactivating Ras and thereby fueling the cancer’s propagation and invasion. Intriguingly, genetic alterations can also wield an indirect influence on cancer plasticity. For example, the loss of the Rb1 gene precipitates a cascade of epigenetic disruptions in retinoblastoma [140]. Furthermore, the genetic landscape of cancer cells often sculpts the tumor microenvironment phenotype observed in many solid tumors, with modifications in environmental factors reciprocally impacting cancer cell behavior [141]. Understanding these intrinsic genetic shifts is paramount, as they form the bedrock upon which cancer develops, diversifies, and adapts, continually challenging therapeutic efforts.

#### Epigenetic changes

Initiatives exploring the cancer genome have serendipitously underscored the significant role of epigenetic modifications in the genesis and plasticity of cancer. Large-scale investigations into cancer genomes revealed a striking observation: chromatin regulatory proteins are mutated in about 50% of human malignancies, a discovery that was not the primary focus of these studies [142]. Furthermore, cancer cells often exhibit genome-wide alterations in methylation patterns, indicative of profound epigenetic disturbances. Epigenetics, defined as heritable changes in gene expression that do not involve alterations to the underlying DNA sequence, emerges as a crucial conductor of cellular plasticity within cancer. These modifications navigate the intricate interplay between environmental stimuli and gene expression control, essentially by rendering chromatin more accessible or more compact [134]. Dysregulation in epigenetic control can result in stalled differentiation or aberrant cell reprogramming, creating an excessively open or overly restrictive chromatin landscape. Intriguingly, cancer genomes have unveiled the re-emergence of a bivalent chromatin state—characterized by both active (H3K4me3) and repressive (H3K27me3) marks near gene promoters—a state reminiscent of what occurs during embryonic development when chromatin is primed and poised for transcription in rapidly dividing and differentiating tissues [134]. This bivalency usually resolves as cells reach their terminal differentiation in normal developmental processes. However, in cancer stem cells, a perpetual bivalent state can endow the cell with a remarkable ability for continuous self-renewal and phenotypic switching, thereby promoting relentless growth and propagation [143].

Epigenetic reconfigurations can take various forms, including chromatin remodeling, as orchestrated by the SWI/SNF complex, and covalent histone modifications, such as methylation and acetylation. These epigenetic shifts can be triggered by mutations or nonmutagenic factors, contributing to a dynamic chromatin environment [144]. Several components involved in these processes, including those responsible for methylation (e.g., DNMT1, DNMT3A), demethylation (e.g., TET family), chromatin remodeling (e.g., ARID1A, ARID1B, ARID2), insulation (e.g., CTCF, cohesin), histone modification (e.g., H3.3, ATRX, DAXX, H3F3A, HIST1H3B, HIST1H1C), and others involved in repressive (e.g., EZH2) or active (e.g., HAT, KDM) chromatin states, have been implicated in cancer’s complex epigenetic narrative [142, 145].

A common epigenetic hallmark in cancer is the hypermethylation of CpG islands in promoter regions of tumor suppressor genes, coinciding with a global hypomethylation landscape that may benefit oncogenes. This phenomenon was first documented in the promoter region of the retinoblastoma gene and was subsequently identified in various other cancers, including renal cell carcinoma with hypermethylation of the VHL gene promoter [146]. Similar aberrant methylation landscapes are observable in cases like BRCA-mutated breast cancer and MMR-mutated colon cancer. Advancements in understanding chromatin topology within cancer cell nuclei have unveiled extensive reorganizations at multiple genomic levels. One mechanism of note involves CTCF, an insulator protein often disrupted by DNA hypermethylation. CTCF and cohesins typically demarcate topologically associated domains (TADs), which are critical regulatory units conserved in their transcriptional activities. When these domains are perturbed, particularly through CTCF hypermethylation, their regulatory capacity diminishes. CTCF functions as a crucial insulator by preventing inappropriate interactions between enhancers and nontarget promoters, thereby maintaining the integrity of chromatin architecture and gene expression profiles [134, 147]. In sum, the subtle yet profound nature of epigenetic changes orchestrates a complex layer of control in cancer development and progression, influencing cellular identity, behavior, and plasticity. Their comprehensive understanding is pivotal for unraveling the intricacies of cancer and potentially harnessing these modifications for therapeutic advantage.

#### Chromosomal instability: a catalyst for phenotypic plasticity and resistance

A hallmark of most tumor cells is their pervasive genomic instability, a trait closely associated with increased resistance to treatment protocols. At the heart of maintaining genomic integrity is the DNA damage response (DDR), an intricate signaling network that facilitates DNA repair, orchestrates cell cycle checkpoints, activates apoptotic pathways, and engages the innate immune response. However, when key components of this network, such as the p53 pathway and various DDR genes, accumulate mutations, the result is bypassing the senescence-associated checkpoint, setting the stage for rampant genomic instability. This instability manifests as a spectrum of structural and numerical chromosomal aberrations, a phenomenon recognized as chromosomal instability (CIN) [148]. CIN is not merely a byproduct of cancer progression; it actively contributes to the disease’s aggressiveness and complexity. Factors such as cytotoxic chemotherapy, errors during mitosis, and replication stress are known contributors to the genesis of CIN. Notably, cancers that exhibit high degrees of CIN tend to be more aggressive, metastasize more readily, and show greater resistance to treatment.

The relationship between CIN and cancer’s evolutionary trajectory might be partially explained by the activation of the cGAS/STING pathway and the resulting increase in karyotypic diversity. With the advent of whole-genome sequencing, our ability to assess these karyotypic aberrations within tumor cells has dramatically improved. This technological advance has facilitated the development of models that simulate the birth and progression of chromosomally unstable tumors. One such model suggests that the early loss of tumor suppressor genes allows cells to tolerate whole genome duplication (WGD). This is followed by karyotypic pruning, a process that refines the chromosomal landscape to yield karyotypes that confer survival advantages, often resulting in near triploid cells.

CIN is not a static event; it is an ongoing process that shapes both primary and metastatic tumors. This continuous reshuffling results in subclonal somatic copy number alterations (SCNAs), which are evident in various cancer stages. Under the relentless pressure of selection forces, cancer cell populations undergo subclonal loss of heterozygosity (LOH), and gene amplifications emerge through several mechanisms. These include chromosomal breakage-fusion-bridge (BFB) cycles and a phenomenon known as chromothripsis, a dramatic restructuring of chromosomes. The chaos of CIN also gives rise to various unique chromosomal structures, such as micronuclei (MN), fold-back inversions, extrachromosomal DNA (ecDNA), and homogenously staining regions (HSRs), each potentially playing a role in cancer’s adaptability and resistance [149]. In conclusion, chromosomal instability is a driving force behind the phenotypic plasticity and resilience of cancer cells. By fostering a diverse population of cells with varying genetic makeups, CIN ensures that some cells will survive, even under adverse conditions such as treatment, thereby perpetuating the disease’s progression and complicating therapeutic intervention. Understanding the mechanisms underlying CIN is crucial for developing strategies to predict, combat, and potentially exploit this instability for therapeutic purposes.

## Tumor heterogeneity and cancer cell plasticity

This section delves deeper into the labyrinth of oncological complexity, highlighting the pivotal interplay between tumor heterogeneity and cancer cell plasticity. This relationship is crucial, underscoring how the diverse cellular environments within tumors contribute to the adaptability and resilience of cancer cells. The insights gathered in this section form a critical nexus in our broader understanding, establishing a foundation upon which subsequent sections will build.

### The dual facets of tumor heterogeneity: intratumor and intertumor heterogeneity

Cancer, a notorious entity, thrives on diversity and adaptation. This principle is evident in the phenotypic and functional heterogeneity exhibited by cancer cells within the same tumor, owing to a confluence of genetic mutations, environmental influences, and reversible cellular changes [150]. One intriguing aspect of this heterogeneity is observed in certain cancers where tumorigenic cancer stem cells differentiate into non-tumorigenic progeny, hinting at a hierarchical structure. However, the extent to which the stem cell model is applicable across various cancers and its implications for clinical behavior remains elusive. Advanced techniques like lineage tracing and deep sequencing promise to shed light on these mysteries, potentially redefining our understanding of treatment resistance and disease progression. The grim reality is that over 90% of cancer mortalities are attributed to recurrence and metastasis, underscoring the disease’s insidious ability to re-emerge locally or colonize distant bodily sites. Treatment options for such advanced stages are tragically limited, largely due to the formidable adaptability of cancer cells—their knack for morphing into forms defiant to therapy. Enhancing patient survival hinges on unraveling the biological intricacies governing this cellular flexibility. Key drivers behind this relentless adaptability are epigenetic reprogramming and the influences of the tumor microenvironment, both of which fuel the dynamism integral to tumor heterogeneity and cancer cell plasticity.

This heterogeneity is two-pronged: intratumoral and intertumoral. Intratumoral heterogeneity refers to the diverse population of cancer cells coexisting within a single tumor. In contrast, intertumoral heterogeneity denotes the genetic discrepancies among different patients bearing the same type of tumor [150]. The former is particularly insidious, arising from a mix of genetic mutations, variations in gene expression, cellular state transitions, and environmental shifts, creating a breeding ground for cancer propagation and therapeutic resistance [151].

Historically, two models—the “clonal evolution” (CE) model and the “cancer stem-like cell” (CSC) model—have been proposed to elucidate intratumor heterogeneity. The CE model suggests a Darwinian approach, where random genetic alterations provide certain clones a competitive edge for survival [152, 153]. Conversely, the CSC model focuses on a minute faction of cells endowed with self-renewal capabilities that initiate and sustain tumors [144, 154, 155]. Emerging from these is a hybrid concept, “CSC plasticity,” positing that CSCs can oscillate between stem and differentiated states, influenced by both intrinsic genetic and extrinsic environmental cues.

One such environmental cue is the EMT, a process known to augment tumor-initiating potentials. Interestingly, CSCs seem to have an amplified EMT program, suggesting that EMT traits are intertwined with CSC properties [46, 156, 157]. This dynamic interplay between CSCs and EMT is further modulated by the tumor microenvironment, replete with elements like growth factors, cytokines, CAFs, tumor-associated macrophages (TAMs), and hypoxic conditions, alongside intrinsic factors like genetic mutations and epigenetic shifts [158, 159]. This nexus not only fosters therapeutic resistance and metastatic spread but also underpins disease recurrence.

Evidence points to the co-expression of epithelial and mesenchymal genes bolstering stemness in cancer cells, evidenced by the formation of “tumor-spheres” [160]. Additionally, studies in prostate cancer models indicate that cells in a partial EMT state may possess enhanced tumor-initiating capacities akin to fully mesenchymal cells [161].

This suggests a potential link between cancer cell stemness and a partial EMT state, challenging the traditional dichotomy of the CE and CSC models and proposing a more fluid, integrated understanding of cancer heterogeneity.

This fluidity is further supported by studies showing non-stem cancer cells’ ability to acquire stem-like traits, a transformation driven by factors like ZEB1, a key EMT player [162]. CSCs, identified mainly through cell surface markers, display remarkable heterogeneity and adaptability across various cancers. These markers, however, are not set in stone; they can vary within a single tumor type and across patients, reflecting the tumor’s genetic landscape [163].

For instance, in glioblastoma, multiple markers have been employed to identify stem cells, but their reliability is contentious, highlighting the inherent plasticity within CSC populations [164].

Colorectal cancer research further underscores CSC plasticity’s role in tumor progression. LGR5, a marker for colorectal CSCs and a Wnt target gene, highlights this plasticity. LGR5 + cells can revert to LGR5 − under drug treatment, and both populations can regenerate the tumor *in vivo* [165]. Recent studies have even identified specific markers for metastatic CSCs, adding another layer of complexity [166]. In conclusion, tumor heterogeneity and cancer cell plasticity are intricate, dynamic, and integral to the cancer paradigm. They complicate treatment strategies but also offer a rich tapestry of targets for future therapies. Understanding this variability and adaptability, especially the plasticity within CSCs and their interaction with the microenvironment, is crucial in our quest to outsmart cancer at its own game.

#### Impact of tumor heterogeneity on diagnosis and treatment

Tumor heterogeneity poses a formidable challenge in cancer management, significantly influencing diagnostic precision and treatment efficacy. As our comprehension of cancer’s molecular landscape has deepened, thanks to transformative research approaches in cancer therapeutics, it is become clear that the path to effective treatment is obstructed by the complexity within and between tumors. This diversity, a hallmark of cancer biology, manifests in genetic, phenotypic, and functional disparities among cancer cells within a single tumor (intratumoral) or across different tumors in the same individual or different individuals (intertumoral).

In patients with advanced malignancies, the initial promise of targeted therapies often fades as treatment resistance emerges, culminating in disease progression and metastasis. This resistance is a multifaceted phenomenon: the evolutionary nature of tumors means that cancer cells continually acquire new molecular aberrations, fostering the growth of subclones that can evade the current therapy. The monotherapeutic approaches that dominate the treatment of advanced-stage cancers tend to fall short because they do not address the entirety of this heterogeneity, leading to suboptimal outcomes.

Traditional methods of molecular diagnostics, primarily reliant on tumor tissue biopsies, are insufficient for real-time tracking of these evolving cancer landscapes. The advent of novel techniques, such as the analysis of circulating cell-free DNA in plasma, offers a minimally invasive avenue to monitor the molecular alterations in cancer, potentially facilitating timely and personalized therapeutic interventions [167, 168].

However, this innovation does not overshadow the challenges posed by intratumor heterogeneity. A key hurdle in personalized medicine is that genetic variability within a single tumor can lead to the emergence of therapy-resistant clones. The standard practice of using formalin-fixed, paraffin-embedded samples from initial biopsies or surgeries for genomic analysis might not capture the full spectrum of genomic alterations, especially those acquired in response to therapy or during metastasis. Different metastatic sites within the same patient might harbor unique genetic profiles, adding another layer of complexity [169].

A landmark study by Gerlinger et al. [170] exemplified this complexity by elucidating the distinct molecular characteristics across multiple primary and metastatic sites in renal carcinoma. The study gave rise to the “tree and branches” model of cancer evolution, highlighting the divergent paths that tumors can take as they develop and metastasize. The implication is profound: the therapeutic strategy for advanced cancers needs a paradigm shift to accommodate this diversity and fluidity.

The TME further complicates this scenario. Components of the TME, such as cytokines and other extracellular matrix constituents, have been shown to contribute to tumor cell survival and therapy resistance. For example, certain chemotherapies induce the release of pro-inflammatory cytokines like interleukin-6 in lymphoma models, fostering the creation of “chemo-resistant niches” that protect cancer cells [171].

Considering cancer through a Darwinian lens, the disease evolves by natural selection, where random genetic and epigenetic changes confer survival advantages to certain clones. This leads to a spectrum of cancers, from simple clonal malignancies with minimal heterogeneity (e.g., BCR-ABL-positive CML) to highly complex mosaic cancers with extensive differences between subclones [172–174]. The latter, rife with intratumoral heterogeneity, presents the most significant challenge, often resisting both targeted and broad-spectrum therapies. Addressing tumor heterogeneity demands a more nuanced approach than current standards provide. It calls for real-time, comprehensive molecular profiling and a multifaceted treatment strategy that considers the dynamic and complex nature of cancer. Only by embracing the depth of cancer’s biological complexity can we hope to improve outcomes in this relentless disease.

## Drug resistance and cancer cell plasticity

In the realm of oncology, while numerous therapeutic advancements have significantly improved patient survival rates and enhanced quality of life, the persistent challenge of therapy-induced drug resistance looms large, often undermining the efficacy of treatment protocols. Alarmingly, resistance to cancer drugs is implicated in approximately 90% of mortality cases associated with the disease [175]. This phenomenon is particularly pronounced in the context of targeted therapies, where the emergence of acquired drug resistance is intimately tied to the remarkable plasticity of tumor cells—a form of biological “hide-and-seek” that enables cancerous cells to adapt, survive, and proliferate. Contrary to traditional belief, drug resistance in cancer is increasingly recognized as a dynamic, often reversible state rather than a fixed genetic alteration [176].

This adaptability underscores the concept of cancer cell plasticity, the cells’ innate ability to rapidly alter their phenotypic and molecular profiles in response to environmental pressures, such as drug exposure. Such plasticity confers a survival advantage, allowing cancer cells to navigate the pharmacological landscape and developing resistance to a spectrum of therapeutic agents. This malleable nature of cancer cells calls for an in-depth understanding of the molecular mechanisms steering this induced plasticity. Unraveling these intricate processes is fundamental to devising innovative therapeutic strategies. It necessitates a shift in focus from a static view of cancer genetics to a more dynamic understanding of genomic variability and adaptability within tumor populations.

Future research endeavors should prioritize the exploration of this adaptive landscape, dissecting the cellular pathways and molecular dialogs involved in therapy-induced resistance. By illuminating the molecular contours of cancer cell plasticity, we pave the way for developing next-generation therapeutics. These novel interventions, designed to outsmart the cunning adaptability of cancer cells, hold the promise of transforming the prognosis for patients, turning the tide in our ongoing battle against this relentless disease.

### EMT and drug resistance

The phenomenon of developmental program reactivation stands as a pivotal mechanism governing various adult disease trajectories, notably influencing EMT pathways integral to the development of drug resistance. Intensive research endeavors have sought to elucidate the relationship between gene and protein expression profiles in tumor tissues and the consequent clinical outcomes in cancer patients. Amidst debates, a consistent observation has been the correlation between heightened expression of mesenchymal and stromal markers and a pronounced resistance to various treatments, spanning chemotherapy, targeted therapy, radiation, and immunotherapy. In a landmark study, Farmer et al. [177] identified a compelling association between the upregulation of genes within the stromal metagene and increased resistance to neoadjuvant chemotherapy—specifically regimes of 5-fluorouracil, epirubicin, and cyclophosphamide—in patients with estrogen receptor (ER)-negative breast cancer [178]. Similarly, rigorous gene expression and proteomic profiling analyses have led to the formulation of a robust 76-gene mesenchymal signature. This signature has proven instrumental in predicting the onset of resistance to treatments with EGFR-TKIs and PI3K/Akt inhibitors in various non-small cell lung cancer (NSCLC) cell lines and clinical specimens. This finding underscores the profound impact of the phenotypic spectrum (specifically, epithelial versus mesenchymal states) on the efficacy of drug responses [179–182].

Expanding the scope beyond genetic markers, proteomic analyses have enabled the differentiation between MAPK inhibitor–resistant and MAPK inhibitor–sensitive melanoma cells. Notably, two markers, PTRF and IGFBP7, have been associated with a phenotypic shift from a melanocytic to a mesenchymal state, offering new avenues for identifying drug-resistant profiles. In summation, the evidence marshaled above lends substantial credence to the proposition that EMT programs are intricately involved in mediating resistance to both cytotoxic and targeted therapeutic agents across various cancer forms. This insight not only underscores the complexity of therapeutic resistance but also signifies the need for a nuanced understanding of cellular plasticity and reprogramming in effective cancer treatment strategies [183].

The advent of immune checkpoint blockade (ICB) therapies, particularly those targeting cytotoxic T lymphocyte–associated protein 4 (CTLA-4) and the programmed cell death 1/programmed cell death ligand 1 (PD-1/PD-L1) pathways, has marked a transformative era in the clinical management of various advanced cancers. However, the enthusiasm for these novel therapies is tempered by significant hurdles in their application across multiple cancer types, including lung adenocarcinoma [184, 185], melanoma [186], and pancreatic malignancies [187]. Specifically, the issues of suboptimal response rates, resistance to immunotherapy, and instances of delayed recurrence continue to cast shadows on the field of cancer immunotherapy.

The role of EMT programs in influencing the efficacy of immunotherapies is a topic of ongoing debate, with the underlying molecular mechanisms governing immune evasion yet to be fully discerned. Certain investigations have identified a positive correlation between the presence of EMT-related markers and enhanced T cell infiltration, implying an augmented sensitivity to ICB therapies. Conversely, there is emerging evidence indicating that tumors characterized by the expression of EMT or stromal-associated genes frequently correlate with more dire clinical prognoses, manifesting in diminished progression-free and overall survival rates [188, 189]. These contrasting observations suggest a complex interplay between EMT programs and the tumor immune microenvironment. Understanding these dynamics is crucial, as they not only impact the response to current immunotherapies but also provide pivotal insights for the development of future therapeutic strategies aimed at circumventing resistance and improving patient outcomes in the realm of cancer treatment.

### Molecular mechanisms underlying drug-induced cancer cell plasticity

The intricacies of how pharmaceutical agents influence cellular plasticity on a molecular scale remain somewhat enigmatic. However, it is hypothesized that this involves a nuanced, multistage transformation where cancer cells initially adopt a quiescent, drug-tolerant phase, subsequently undergoing a progressive reconfiguration toward a drug-resistant phenotype. This concept of drug-tolerant persisters, extensively studied *in vitro*, offers insights into the survival tactics employed by slow-cycling cells [190].

#### The phenomenon of slow-cycling (dormant) cancer cells

Phenotypic plasticity demonstrated first in bacteria involves a strategic shift between a drug-sensitive, proliferative state and a drug-tolerant, slow-cycling one [191, 192]. Remarkably, bacteria surviving antibiotic treatment can revert to a drug-sensitive, proliferative state upon cessation of the drug, indicating that these adaptable shifts are not due to genetic alterations but are more likely due to phenotypic flexibility. A parallel survival strategy has been observed in cancer cells subjected to *in vitro* treatment; here, cells termed “persister” prefer transient, reversible changes over potentially detrimental permanent genetic mutations, optimizing population fitness. Initially discerned in non-small cell lung cancer, these drug-tolerant persisters (DTPs) are specialized cells that temporarily endure lethal drug concentrations, with analogous subsets identified in glioblastoma [193], melanoma [190], and colon cancer [194]. Recent findings by Shaffer et al. [176] highlight that certain melanoma cells, characterized by a transient upregulation of resistance genes like AXL before drug intervention, exhibit enhanced survival prospects compared to their parental counterparts. Intriguingly, this drug-resistant state is not genetically transmissible, suggesting DTPs originate from dynamic alterations within the cell collective rather than a fixed subpopulation [176].

It is compelling to acknowledge the presence of these drug-tolerant, slow-cycling cells in *in vivo* contexts. For instance, residual cells in basal cell carcinoma (BCC) displaying these traits can instigate relapse post-therapy cessation [195, 196]. Their successful elimination of post-stimulated proliferation under experimental conditions indicates a necessity for cellular quiescence in acquiring drug tolerance [196]. Moreover, a quiescent state resembling nutrient deprivation appears pivotal during the initial stages of minimal residual disease (MRD) in melanoma. However, while quiescence is essential for drug tolerance, it does not restrict the heterogeneity of subsequent resistance mechanisms, as demonstrated in coexisting drug-tolerant phenotypes [197].

Interestingly, despite the temporary nature of this state, various resistance strategies, including irreversible genetic alterations commonly observed in clinical specimens, can manifest during extended DTP cultures [198]. Thus, under continual drug pressure, cancer cells may experience a sequential metamorphosis, beginning with a reversible transcriptomic overhaul to attain a slow-cycling state, followed by resumption of proliferation, and culminating in enduring resistance due to additional epigenetic shifts or genetic modifications reactivating the targeted pathways. This slow-cycling, drug-tolerant phase seems to be a universal therapeutic bypass, irrespective of cancer type or specific treatment, thus presenting itself as an attractive target for therapeutic intervention [198].

### Emergence of altered cellular identity

Cancer cells exposed to pharmacological agents can undergo extensive reprogramming, shifting their identity to develop drug tolerance. This metamorphosis is associated with epigenetic and transcriptional modifications, consistent with the reported reversibility of phenotypic transitions. The genesis of a novel cellular identity post-treatment may arise from an intricate interplay between the tumor microenvironment and the original cancer cell lineage.

The transient character of these identity shifts implies a significant role in epigenetic reprogramming, standing in contrast to genetic alterations that would induce an unchangeable transformation. In support of this, research indicates that such phenotypic oscillations are frequently accompanied by profound modifications in histone methylation landscapes, attributable to the perturbation of epigenetic modulators [199–203].

### The role of transcription factors in modulating cellular plasticity

Investigating cellular adaptability in response to drug interventions heavily relies on transcriptional profiling, an indispensable component within the research toolkit. This approach has illuminated the pivotal function of various essential reprogramming and lineage-specific transcription factors, orchestrating cellular plasticity and facilitating evasion from treatment through lineage conversion. Notably, the SOX gene family, encoding a group of transcription factors, emerges as a critical conductor in determining cell fate. Their substantial involvement in regulating cellular plasticity has been documented across multiple cancer forms [204].

### Influence of signaling pathways on cellular plasticity

Several signaling cascades intricately shape the landscape of drug-induced cellular plasticity. For instance, in castration-resistant prostate cancer (CRPC), there is a notable dysregulation of the WNT-β-catenin pathway. This aberration not only promotes resistance to androgen depletion but also fosters the acquisition of neuroendocrine characteristics, contributing to the complexity of treatment resistance. A similar mechanism is observed in BCC, where WNT signaling steers drug tolerance by inducing an intermediate filament epithelial (IFE)-like state that operates independently of the Hedgehog signaling pathway. Given this context, the pharmacological curtailment of WNT signaling has shown promising therapeutic potential. Interventions employing the porcupine inhibitor LGK-974, which obstructs the activity of porcupine acyltransferase essential for WNT secretion, or antibodies targeting the WNT receptor LRP6, have demonstrated efficacy. These strategies effectively reduced residual tumor burdens and delayed tumor resurgence post-vismodegib therapy withdrawal. Impressively, the extent of these therapeutic benefits directly mirrored the degree of WNT pathway suppression, underscoring the pathway’s central role in cellular plasticity and cancer relapse [195, 196, 205–207].

### Strategies for overcoming drug resistance

**Fig. 4:**
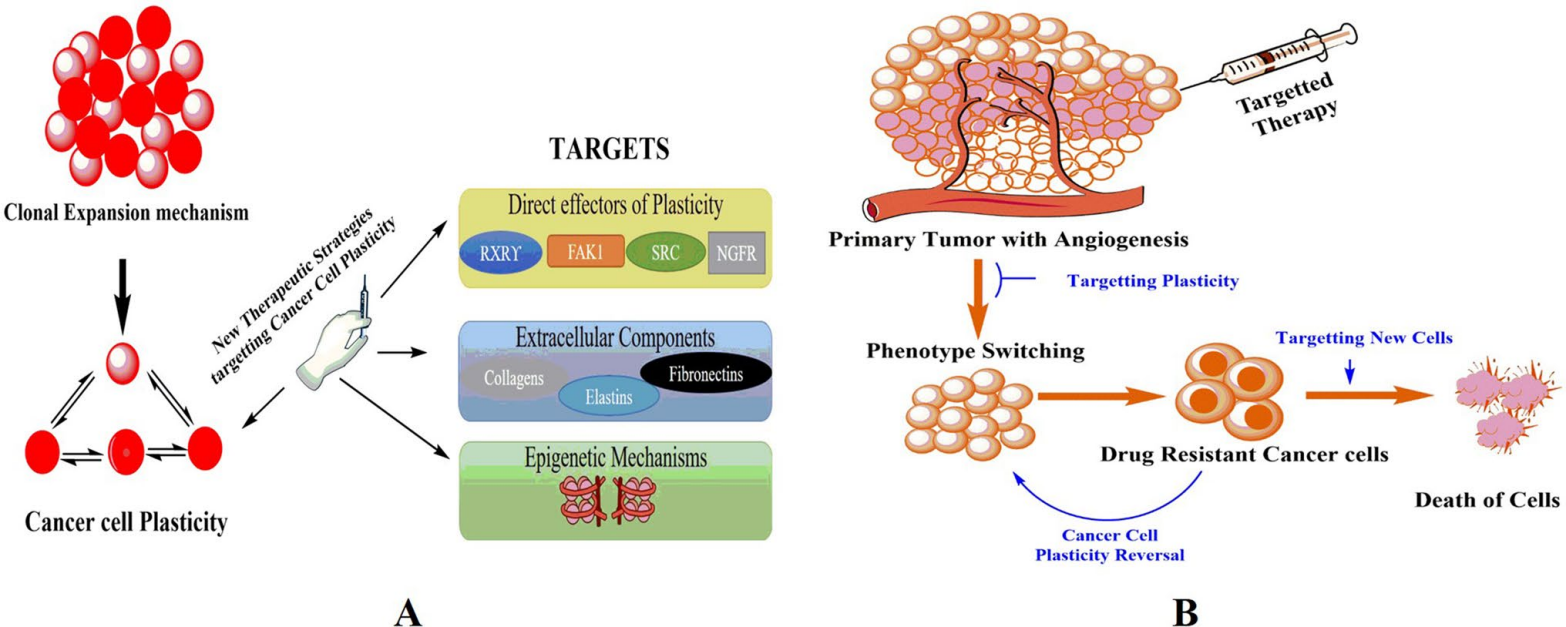
**A** Exploring the origins of cellular plasticity and innovative therapeutic strategies. This figure delves into the intricate origins of cellular plasticity, providing a comprehensive visualization of the multifactorial mechanisms that contribute to this phenomenon. It maps out the path from initial cellular changes to the manifestation of plasticity, detailing the genetic, epigenetic, and environmental factors at play. The figure concurrently showcases emerging therapeutic strategies that precisely target cellular plasticity, highlighting their modes of action, potential benefits, and associated challenges. **B** Comprehensive overview of therapeutic approaches targeting tumor cell adaptability. This figure provides a comprehensive, visually engaging overview of the innovative therapeutic approaches that strategically target the adaptability of tumor cells. The figure details three primary, combinable approaches for effectively addressing tumor cell plasticity. The first approach elaborated upon is the prevention of tumor cell plasticity, outlining potential methods and strategies for this preventive action. The second approach discussed is the reversal of phenotypic switching, which offers insight into the mechanisms that can revert the altered phenotypes to their original states. The third focus is centering therapy on the induced therapy-resistant tumor cells, with the figure delineating the prospective therapies and their targeted actions

The effectiveness of targeted cancer therapies is often undermined by multifaceted resistance mechanisms. Beyond genetic mutations conferring resistance, cancer cells engage in reversible processes that foster a state of drug tolerance. These cells can transition into a slow-cycling, drug-tolerant phenotype, independent of the initially targeted pathway, potentially reverting to drug sensitivity upon treatment cessation or, conversely, evolving sustainable resistance—culminating in treatment relapse. The phenomenon of cellular plasticity has emerged as a critical survival tactic across various cancers, including melanoma, basal cell carcinoma, and prostate and lung adenocarcinomas. Insights into chromatin remodeling and reprogramming factors have gained prominence in decoding the intricacies of this phenotypic adaptability. As delineated in Fig. 4A, unraveling the molecular intricacies of tumor cell plasticity might pave the way for innovative therapeutic strategies. When synchronized with existing anticancer regimens, these novel approaches promise to elicit more profound and durable clinical outcomes [138].

#### Targeting cancer cell adaptability

Strategies zeroing in on cellular plasticity processes—key contributors to drug tolerance and resistance—might usher in more substantial and enduring therapeutic responses. This entails a tri-pronged approach: preemptively curtailing plasticity by inhibiting its regulatory molecular mechanisms, focusing on cells’ post-identity alteration, and reversing the phenotypic transition. Translating these insights into *in vivo* applications remains critical, given our understanding predominantly stems from *in vitro* studies. The potential for adverse effects warrants caution, especially since pathways integral to plasticity, like epigenetic regulators and certain signaling components, also play pivotal roles in physiological homeostasis and repair [138, 208, 209].

#### Inhibiting cancer cell plasticity

A spectrum of strategies could forestall phenotypic switching, including intermittent dosing schedules, concurrent suppression of pivotal pathways reactivated downstream of oncogenic drivers (via integrating targeted therapy with chemotherapy, radiotherapy, or immunotherapy), and specifically targeting slow-cycling cells while blocking signaling pathways crucial for the emergence of new cellular identities [208, 210].

#### Intermittent therapeutic regimens

Adopting combined and intermittent treatment protocols, as opposed to continuous monotherapy, may curtail the development of resistance. Prolonged exposure to treatments can push remaining drug-tolerant cells toward an irreversibly resistant state. Notably, an intermittent dosing strategy extended the response duration in melanoma cells to vemurafenib [211]. However, the complexity of resistance mechanisms, potentially co-emerging, poses significant challenges in crafting intermittent dosing strategies for clinical application [197].

#### Disrupting phenotypic switching with a focus on critical junctions

Slow-cycling, drug-tolerant cells represent a pivotal stage in the continuum of phenotypic switching, as established earlier. Various mechanisms underpin the ability of these cells to withstand drug therapies *in vitro*, encompassing stimulated IGF1R signaling [212], amplified drug efflux [190], endoplasmic reticulum stress signaling, and intricate chromatin remodeling [212, 213].

Notably, the formation of DTPs was thwarted when the NSCLC cell line PC9 underwent simultaneous treatment with EGFR-TKIs and AEW541, a specialized IGF1R kinase inhibitor.

One particularly compelling approach lies in devising agents that target epigenetic regulators, given the profound connection between specific epigenetic disruptions and the emergence of DTPs. For instance, in NSCLC, heterochromatin formation, driven by the trimethylation of lysine 9 on histone H3 (H3K9me3), effectively silences long interspersed nuclear element 1 (LINE-1) repeats. This epigenetic modification dampens the expression of interferon-stimulated genes and antiviral response elements, thereby fortifying DTP survival [214]. Remarkably, drug-tolerant cell populations can be eradicated through the reactivation of LINE-1 elements, achieved by HDAC inhibition using agents like trichostatin A or entinostat (MS-275), as summarized in Table 1. However, the application of trichostatin A in slow-cycling, drug-tolerant melanoma cells elicited only transient responsiveness. Furthermore, the crucial histone demethylases KDM5A/B and KDM6A/B emerge as significant determinants in the sustenance of DTPs. Beyond this, intervening in the signaling pathways essential for establishing new cellular identities could potentially impede the evolution of MRD and reduce the probability of subsequent resistance. This principle is exemplified in ongoing research targeting neuroendocrine transdifferentiation in prostate cancer. Specifically, the implementation of siltuximab, an anti-IL-6 monoclonal antibody, and ruxolitinib, a JAK kinase inhibitor operating upstream of STAT, has been shown to hinder the development of an androgen receptor (AR)-independent, neuroendocrine-like phenotype in both human prostate cancer cell lines and murine models [215]. This strategy underscores the potential of intercepting cellular plasticity at critical junctures, thereby diluting the seeds of drug resistance.

**Table 1:** Drugs and targets for preventing cell plasticity

Therapeutic intervention (single/in combination	Clinical status of drug	Clinical outcome	Indications	Ref
Histone deacetylases				
Vorinostat and bicalutamide	Phase II	All patients have evidence of prostate disease at the time of surgery	Localized prostate cancer	[220]
Vorinostat and gefitinib	Phase I/II	Patients with non-selected NSCLC tolerated the treatment well; however, the PFS did not improve	Progressive NSCLC with relapse or resistance	[221]
Nanatinostat	Phase I	Positive toxicological profile	Progressive solid tumors	[222]
Panobinostat and bicalutamide	Phase I/II	Improved progression-free survival	Castration-resistant prostate cancer (CRPC)	[223]
Vorinostat	Phase I	Recruiting	Melanoma generally resistant to BRAF/MEK inhibitors	[224]
Vorinostat and erlotinib	Phase I/II	There is no significant action in the erlotinib-resistant group	EGFR-mutant NSCLC with relapse	[225]
Bromodomain and extraterminal (BET) domain				
BI894999	Phase Ia/Ib	Recruiting	Hematological disorders and progressive solid tumors	[226, 227]
PLX2853	Phase Ia/IIb	Recruiting	Hematological disorders and progressive solid tumors	[227]
RG6146	Phase I	Patients with diffuse large B cell lymphoma (DLBCL) have a tolerable safety profile	Hematological disorders and progressive solid tumors	[228]
Cyclin-dependent kinase CDK7/12				
ICEC0942	Preclinical	Antitumor efficacy in several cancer model organisms	ER-positive breast cancer	[229]
SY-1365	Phase I	Recruiting	Progressive solid tumors	[230]
Histone demethylases (KDM5 and KDM6)				
YUKA1, CPI-455 (KDM5A-specific), KDOAM-25 (KDM5A-D-specific)	Preclinical	In multiple cancer models, YUKA1 inhibits drug tolerance in EGFR mutant lung cancer treated with gefitinib	NSCLC with EGFR mutation	[231–234]
GSK-J4 (KDM6-specific)	Preclinical	Hinders the proliferation of cells in glioblastoma	Glioblastoma	[193, 235]
Cell signaling mechanism				
IL-6-STAT3 signaling				
Siltuximab and docetaxel	Phase I	CRPC effectiveness when combined with docetaxel	Castration-resistant prostate cancer (CRPC)	[236, 237]
Notch signaling: γ-secretase inhibitor (GSI)				
RO4929097	Phase I	Trials of RO4929097 were prematurely discontinued due to a lack of clinical benefits	Glioblastoma that is persistent or recurrent	[238]
MK0752	Phase I	Weekly dosage was well tolerated, and clinical advantages were seen	Breast cancer, which is progressing	[239]
WNT signaling				
LGK-974	Phase I	Initial findings point to a tolerable safety profile	Malignancies dependent on WNT	[240]
CGX1321	Preclinical	Recruiting	Progressive solid tumors	[241]
Wnt-C59	Preclinical	Mammary tumor progression was stopped in mice without any obvious toxicity	Adenocarcinomas of the breast	[242]
Anti-LRP6	Preclinical	Delayed growth of the tumor	Colorectal cancer	[243, 244]
Tumor necrosis factor (TNF) signaling				
BMS345541, SC-514, and MAPK inhibitor	Preclinical	Increased effectiveness of MAPK inhibitors in melanoma (skin cancer)	Skin cancer	[245]

#### Targeting emergent cellular identities

Concentrating on newly developed drug-tolerant cellular identities presents an alternative avenue to impede cellular plasticity. This could involve employing existing therapies innovatively or uncovering entirely new vulnerabilities within these emergent cells. Ongoing research probes whether neuroendocrine-differentiated tumors share sensitivity profiles with their *de novo* small cell counterparts. For example, transformed small cell lung cancer (SCLC) diverges from its non-transformed EGFR-mutant adenocarcinoma precursors by lacking EGFR expression, thereby rendering them resistant to EGFR inhibitors [216]. However, these transformed SCLCs bear a closer resemblance to *de novo* SCLCs, given their transient responsiveness to platinum-etoposide [53, 55]. Interestingly, a recent retrospective study involving 67 patients indicated that transformed SCLCs respond more favorably to taxanes compared to *de novo* SCLCs but displayed resistance to checkpoint inhibitors, mirroring the behavior of typical EGFR-mutant adenocarcinomas [217].

In the realm of prostate cancer, a phase II trial identified a subset of enzalutamide-resistant patients who exhibited exceptional responses to the anti-PD1 antibody pembrolizumab. Furthermore, the receptor tyrosine kinase AXL, correlated with the emergence of EMT markers in NSCLC31, presents itself as a potential novel target. Encouragingly, co-administration of the EGFR inhibitor erlotinib with the AXL inhibitor SGI-7079 enhanced the sensitivity of mesenchymal-like NSCLC cells to erlotinib in an NSCLC mouse xenograft model, as detailed in Table 2 [218].

**Table 2:** Drugs and targets for the new cell identity

Therapeutic intervention (single/in combination	Clinical status of drug	Clinical outcome	Indications	Ref
AXL (receptor tyrosine kinase)				
SGI-7079/erlotinib	Preclinical	Mesenchymal-like NSCLC cells became more sensitive to erlotinib	Non-small cell lung cancer	[218]
TP-0903	Phase Ia/Ib	Recruiting	Progressive solid tumors	NCT02729298
BGB324/erlotinib	Phase II	Met the initial efficacy objective	Non-small cell lung cancer	NCT02424617
Glutathione peroxidase 4 (GPX4)				
RSL3, ML210	Preclinical	Specific drug-tolerant persister (DTP) eradication	–	[246]

#### Reversing cellular plasticity

Considering that epigenetic mechanisms predominantly govern cellular plasticity, it is plausible to posit that this plasticity can be reversed, thereby re-establishing cellular sensitivity to pharmacological interventions. However, the inhibition of epigenetic regulators critical to cellular plasticity, such as REST, must be approached with caution due to their broad physiological roles. On a promising note, leveraging thalidomide analogs or proteolysis-targeting chimeras (PROTACs) could lead to the effective degradation of vital reprogramming transcription factors, like SOX factors, spurred by recent insights into the potential targetability of these transcription factors, as depicted in Fig. 4B.

An alternative strategy involves obstructing lineage-specific transcription factors by targeting the pertinent chromatin-modifying enzymes, given the intimate relationship between these factors and their chromatin environment. Moreover, extensive efforts are being made to reverse EMT, potentially by inhibiting TGF signaling. Yet, the therapeutic targeting of this pathway is complicated by the multifaceted roles of TGFs in cancer, notably their dichotomous roles across various stages of tumorigenesis, as summarized in Table 3 [219].

**Table 3:** Drugs and targets for the reverse cell identity

Therapeutic intervention (single/in combination	Clinical status of drug	Clinical outcome	Indications	Ref
Enhancer of zeste homolog 2 (EZH2)				
GSK2816126	Phase I	Due to insufficient clinical activity, the trial was discontinued	Solid tumors and DLBCL	NCT02082977
Tazemetostat	Phase I/II	A good safety record and antitumor activity	Progressive solid tumors and B cell lymphoma	NCT01897571 [247]
PF-06821497	Phase I	Recruiting	Castration-resistant prostate cancer (CRPC)	NCT03460977 [248]
Dual HDAC and HMGR inhibitor				
JMF3086	Preclinical	Restores EGFR-TKI sensitivity	Non-small cell lung cancer	[249]

This complexity underscores the need for continued exploration into the strategic application of TGF inhibitors within combination therapies, especially in drug-induced cellular plasticity.

## Conclusion and future directions

Cancer cell plasticity is a pivotal research area, illuminating the intricate tapestry of factors contributing to therapeutic resistance. This research delineates various intrinsic and extrinsic cellular components, collectively steering the phenomena of plasticity and playing a significant role in the emergence of treatment resistance. The unveiling of oncogenic driver mutations has heralded a new era in targeted treatments, promising focused suppression of mutation-induced pathways. These targeted treatments have showcased unparalleled clinical responses compared to traditional chemotherapy. Yet, the elation is tempered by the emergence of resistance mechanisms upon sustained drug exposure, leading to transient and incomplete treatment responses. This predicament has spotlighted the crucial role of cell plasticity as a formidable accomplice in therapeutic evasion beyond the confines of known genetic alterations. The strategic targeting of cellular plasticity stands out as a unique opportunity to enhance the efficacy of existing treatments. Many challenges loom despite the leap in understanding the molecular underpinnings of cell plasticity, including epigenetic and transcriptional alterations and interaction with the tumor microenvironment. The relationship between clinicopathological factors and the propensity of cancer cells for phenotypic alterations remains enigmatic. The urgent need to uncover new predictive biomarkers of cell plasticity and to understand the exact temporal dynamics of phenotypic plasticity during therapeutic interventions is paramount. Recent studies have unmasked the complexity of drug-induced cell plasticity, revealing diverse drug-tolerant states that potentially contribute differently to relapse. These findings emphasize the need for tailored strategies to target remaining cancer cells, considering the MRD composition heterogeneity. Unveiling the interplay between various drug-tolerant states and understanding their role in relapse will be critical for enhancing therapeutic efficacy and improving patient outcomes. Furthermore, the intricate dynamics of cell plasticity in response to drug therapy, particularly highlighted in melanoma studies, underscore its multifaceted nature beyond *in vitro* expectations. Notably, cells transitioning from a temporary drug-tolerant state to irreversible drug resistance add another layer of complexity, demanding comprehensive exploration to unlock novel therapeutic avenues.

In conclusion, while advancements in targeting pathways promoting cellular plasticity have been achieved, a significant enhancement in patient prognosis remains elusive. The forward path necessitates a multifaceted approach, combining robust understanding and innovative strategies to effectively counter the evolving landscape of cancer cell plasticity and therapeutic resistance. Continuous efforts in unraveling the complexities of cellular plasticity and investing in innovative research and technologies stand as crucial steps toward achieving this goal, heralding a new horizon in cancer therapeutics.

## Data Availability

Not applicable.
